# Mapping the SHP2 Allosteric Pocket With Target‐Biased Covalent Fragments

**DOI:** 10.1002/cbic.70310

**Published:** 2026-04-24

**Authors:** Nina‐Louisa Efrém, Noémi Csorba, Machoud Amoussa, Péter Ábrányi‐Balogh, Ziqiong Guo, László Petri, Feng Bo, Vincenzo Di Lorenzo, Yvette Roske, Tibor Viktor Szalai, Levente Mihalovits, József Simon, Jia Li, Oliver Daumke, György M. Keserű, Marc Nazaré

**Affiliations:** ^1^ Medicinal Chemistry Leibniz‐Forschungsinstitut für Molekulare Pharmakologie (FMP) Berlin Germany; ^2^ Karlsruhe Institute of Technology Organic Chemistry I Institute of Organic Chemistry Karlsruhe Germany; ^3^ Medicinal Chemistry Research Group HUN‐REN Research Centre for Natural Sciences (HUN‐REN RCNS) Budapest Hungary; ^4^ National Laboratory for Drug Research and Development HUN‐REN Research Centre for Natural Sciences (HUN‐REN RCNS) Budapest Hungary; ^5^ Department of Organic Chemistry and Technology Faculty of Chemical Technology and Biotechnology Budapest University of Technology and Economics Budapest Hungary; ^6^ Institute of Pharmacy Freie Universität Berlin Berlin Germany; ^7^ Shanghai Institute of Materia Medica (SIMM) Chinese Academy of Sciences Shanghai China; ^8^ Structural Biology Max Delbrück Center for Molecular Medicine in the Helmholtz Association (MDC) Berlin Germany; ^9^ Department of Inorganic and Analytical Chemistry, Faculty of Chemical Technology and Biotechnology Budapest University of Technology and Economics Budapest Hungary; ^10^ Department of Chemistry Organic and Bioorganic Chemistry Bielefeld University Bielefeld Germany

**Keywords:** allosteric inhibition, covalent fragments, phosphatases, SHP2, SuFEx

## Abstract

Targeted covalent inhibitors (TCIs) form covalent bonds with a specific amino acid in their target proteins, offering high selectivity and sustained pharmacologic effects. However, identifying optimal electrophilic warheads and nucleophilic amino acids remains a major hurdle for TCI development. While covalent fragment libraries are efficient in the identification of reactive residues, their inherently weak and transient interactions often fail to address functionally relevant binding sites. Here, we combine the exploratory approach of covalent fragment screening with established inhibitor pharmacophores for covalent mapping of the tunnel allosteric site of the oncogenic phosphatase SHP2. Aryl sulfonyl fluoride (SF) fragments featuring pharmacophore elements to enhance noncovalent interactions (target‐biased fragments) covalently targeted lysine 492 (K492) in the tunnel binding site, while a conventional SF fragment library lacking these features was not reactive toward K492. Covalent engagement of K492 improved enzyme inhibition and provides a starting point for SHP2 TCI development. More broadly, this study underscores how noncovalent interactions direct covalent fragment binding and highlights target‐biased fragments as a complementary strategy to conventional covalent fragment libraries to identify suitable warheads and reactive amino acids in functionally relevant binding sites with minimal a priori knowledge of ligand pharmacophores.

## Graphical abstract

Target‐biased fragments combine electrophilic warheads with inhibitor pharmacophore elements. Applied to the oncogenic phosphatase SHP2, they covalently label K492 in the tunnel allosteric site, enhancing enzyme inhibition and providing a starting point for covalent SHP2 inhibitor development. This approach complements conventional covalent fragment libraries to identify reactive residues in functionally relevant binding sites.

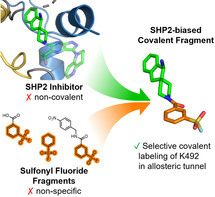

## 1 Introduction

**FIGURE 1:**
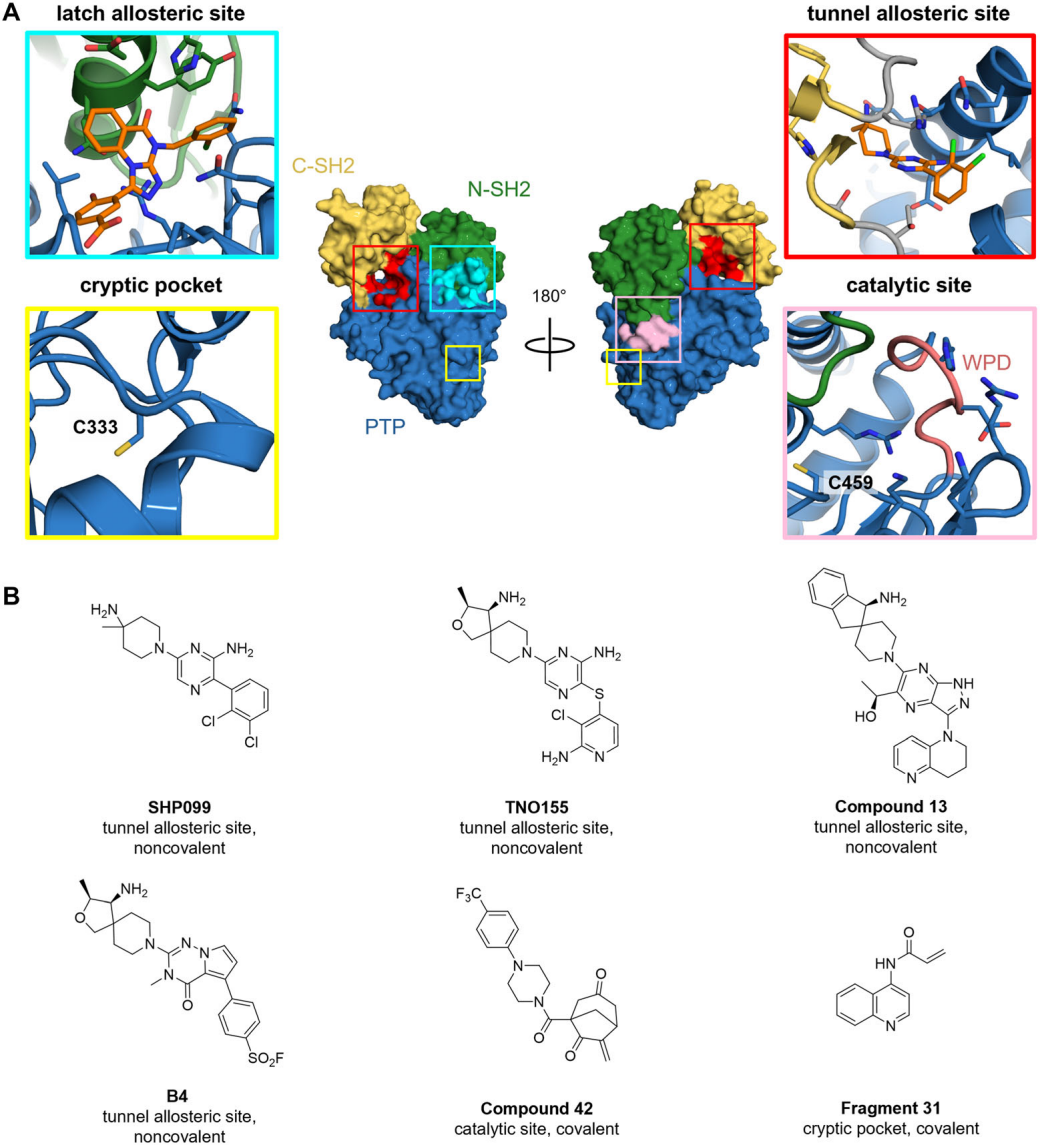
Overview of SHP2 binding sites and chemical structures of allosteric SHP2 inhibitors. (A) Co‐crystal structure of SHP2 in complex with allosteric inhibitors **SHP504** and **SHP099** (inhibitors as orange sticks, PDB ID 6BMW) [6] with annotation of binding pockets reported to accommodate small‐molecule inhibitors. Color coding indicates the location of these pockets on the protein surface as well as the three‐domain architecture of SHP2 (N‐SH2 domain: green; C‐SH2 domain: yellow; catalytic protein tyrosine phosphatase (PTP) domain: blue, interconnecting loops: gray). Cysteine residues that have been targeted by covalent inhibitors are labeled in the close‐up views. Conserved WPD loop of the catalytic site highlighted in salmon. (B) Exemplary structures of noncovalent and covalent allosteric SHP2 inhibitors and fragments: **SHP099** [7, 8], **TNO155** [9], **Compound 13** [10], and **B4** [11] bind to the tunnel allosteric site of SHP2 without covalent bond formation. **Compound 42** [12] and **Fragment 31** [13] bind to the SHP2 PTP domain through covalent engagement of C459 (catalytic site) and C333 (cryptic pocket), respectively.

SHP2 is a cytosolic protein tyrosine phosphatase (PTP) that is encoded by the *PTPN11* gene. It is ubiquitously expressed and plays a key role in oncogenic receptor tyrosine kinase signal transduction and modulation of the tumor microenvironment [1, 2, 3]. Reversible allosteric SHP2 inhibitors with superior selectivity and drug‐like properties compared to previously reported orthosteric site‐targeting inhibitors currently undergo clinical trials, positioning SHP2 as a promising oncology target [4, 5]. While several allosteric pockets have been reported (Figure 1A), SHP2 inhibitors in clinical trials all target the tunnel‐like allosteric site at the interface of the regulatory SH2 domains and the catalytic PTP domain and thereby stabilize the enzyme in an autoinhibited conformation [7, 8, 14, 15]. Despite the leap forward achieved by these allosteric SHP2 inhibitors, activating mutations that occur mainly in developmental diseases and hematologic malignancies [16, 17] disrupt contacts at the SH2‐PTP domain interface and destabilize the autoinhibited conformation of the enzyme, reducing the potency of several reported tunnel‐targeting inhibitors [18, 19, 20].

Targeted covalent inhibitors (TCIs) can maintain inhibitory activity on disease‐related mutated protein isoforms due to their prolonged residence time [21, 22]. Allosteric TCIs combine this advantage of a prolonged pharmacological effect with the enhanced specificity of allosteric modulators, leading to an even more precise abrogation of aberrant protein function [23, 24]. Crucial for TCI efficacy and selectivity is a subtle balance between noncovalent protein–ligand interactions that precede covalent engagement of the targeted nucleophilic amino acid and warhead reactivity [25, 26]. Apart from conventional cysteine‐reactive electrophiles, latent warheads targeting lysine, tyrosine, tryptophan, histidine, serine/threonine, methionine as well as aspartate and glutamate [27, 28, 29, 30, 31, 32, 33, 34, 35, 36, 37, 38], have greatly expanded the scope of proteins amenable to covalent strategies [39]. Nonetheless, the identification of selective TCIs remains a challenging endeavor. For highly tractable target proteins, known high‐affinity noncovalent ligands can be converted into covalent binders by installing suitable warheads in appropriate positions. However, this often affects noncovalent recognition and conversely, scaffold variations can alter warhead reactivity [40, 41, 42]. Even if structural information on the protein–ligand complex is available, amino acid flexibility, steric effects, the microenvironment of the binding pocket as well as induced fit effects influence amino acid side chain nucleophilicity and can lead to unanticipated reactivities [41, 43].

Screening of covalent fragment libraries [44] has emerged as an orthogonal approach in absence of an obvious starting point for TCI development [45]. They have been used for mapping reactive amino acids in recombinant protein targets [45, 46, 47] and in native biological systems using chemoproteomic approaches [48, 49, 50]. Here, commonly, electrophilic fragments with low structural bias and complexity are applied. Amino acid labeling is therefore driven by the warhead reactivity and an intrinsically limited number of weak and transient noncovalent interactions that may not translate into a pharmacological response [41]. Consequently, liganded amino acids are not necessarily located in functionally relevant binding sites and fragment hits require follow‐up validation in functional assays [42, 48, 49]. In addition, the exploitation of covalent fragment hits as starting points for TCI development is complicated by multiple binding modes and the absence of synthetic handles for fragment growing, which could lead to autoreactivity and fragment decomposition.

In the case of the oncogenic phosphatase SHP2, no drug‐like covalent allosteric inhibitor is available to date. Efforts toward covalent inhibition of SHP2 have so far primarily focused on the PTP domain, mostly relying on Cys‐reactive probes. In their early work, Chio, Lim, and Bishop discovered biarsenic SHP2 inhibitors targeting C333 in a cryptic pocket in the PTP domain and later identified acrylamide fragments, exemplified by **Fragment 31**, that bind to the same C333 residue and inhibit SHP2 with moderate potency (Figure 1) [13, 51]. Recently, Hong, Xi et al. included the SHP2 PTP domain in a broad study to identify PTP catalytic site‐targeting electrophilic warheads [52]. *N‐*Phenyl maleimide, nitrovinylbenzene, arylvinyl sulfonates, and, to a lesser extent, chloroacetamide fragments inhibited SHP2 through binding to the PTP domain. Although this study elegantly identified diverse electrophiles that covalently engage PTPs, the sites of covalent modification in SHP2^PTP^ were not determined and most fragments displayed limited selectivity, in line with an analysis by ter Brake and coworkers [53], who observed unspecific labeling by a set of activity‐based protein profiling probes across various PTPs, including SHP2. In a recently published structure–activity relationship study [11], 5‐arylpyrrolo[2,1‐*f*][1,2,4]triazin‐4(3*H*)‐one‐based allosteric SHP2 inhibitors were equipped with aryl sulfonyl fluoride (SF) warheads, exemplified by **B4** (Figure 1B) to engage K492 in the tunnel‐like pocket of full‐length (SHP2^FL^). However, no covalent adducts were detected by intact mass spectrometry (MS).

Here, we aimed to identify reactive, nucleophilic amino acids that can be harnessed for allosteric SHP2 TCI development beyond Cys, focusing on the tunnel allosteric site targeted by SHP2 inhibitors in clinical trials.

## 2 Results and Discussion

**FIGURE 2:**
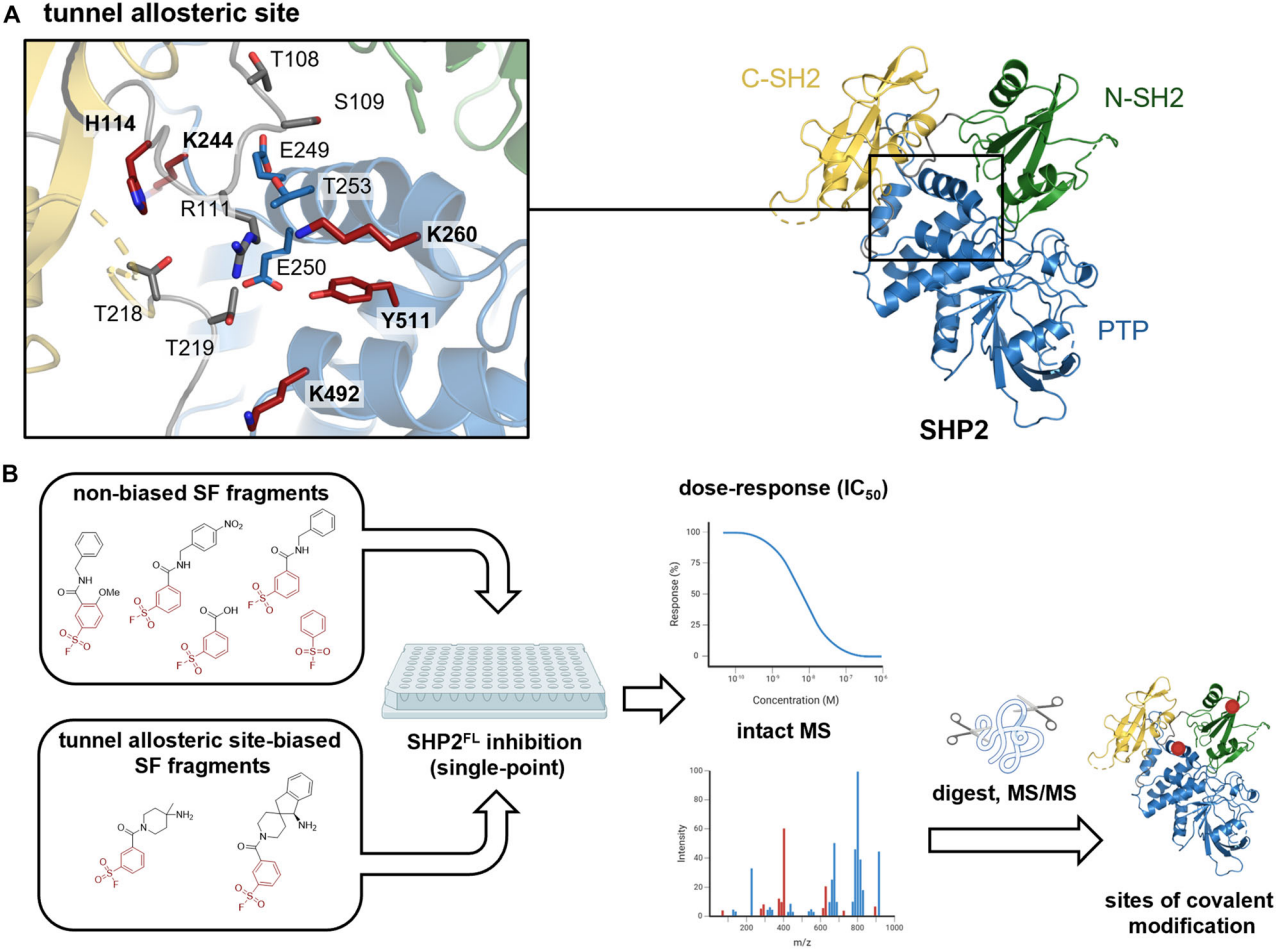
Amino acid layout of the SHP2 tunnel allosteric site and workflow used to identify aryl sulfonyl fluoride (SF)‐reactive residues in SHP2^FL^. (A) Close‐up view of the tunnel allosteric site of SHP2 (PDB ID 8T6G). Cartoon color distinguishes N‐SH2 and C‐SH2 domain (green and yellow, respectively), and the catalytic PTP domain (blue). Interdomain loops are colored gray. Amino acids relevant for inhibitor binding are shown as sticks. Potentially SF‐reactive amino acid residues [54, 55] within a 5 Å radius around the tunnel allosteric binding site of SHP2 are highlighted in red (see also Table S5). (B) To experimentally identify SF‐reactive amino acids in the SHP2 tunnel allosteric site, two sets of SF fragments were investigated: an in‐house, conventional non‐biased SF fragment library and tunnel allosteric site‐biased SFs that contain pharmacophore features for tunnel site targeting. First, fragments were tested for inhibition of full‐length SHP2 (SHP2^FL^) at 100 µM fragment concentration. The most active fragments were characterized in terms of inhibitory potency (IC_50_) and covalent binding to SHP2^FL^ by intact mass spectrometry (MS) followed by identification of the sites of covalent modification by proteolytic digest and tandem mass spectrometry (MS/MS).

To identify reactive amino acid residues in the tunnel allosteric site of SHP2 (Figure 2A) that can be targeted by allosteric TCIs, we started out by mapping ligandable residues in SHP2^FL^ using an in‐house aryl SF fragment library [56]. In a complementary approach, we combined privileged noncovalent recognition elements from reported tunnel allosteric site‐targeting SHP2 inhibitors [57] with the electrophilic SF warhead, giving rise to a set of SHP2 tunnel site‐biased SF fragments. Both fragment sets were initially tested for inhibition of SHP2 at a single concentration. The most active fragments were selected for determination of their half‐maximal inhibitory concentration (IC_50_) and their ability to covalently modify SHP2^FL^ by MS. The sites of covalent modification were subsequently determined by MS/MS following tryptic digestion of SHP2 (Figure 2B, Figure S4).

### 2.1 Non‐biased Fragment Library

We started our investigation by screening of the in‐house SF fragment library against SHP2^FL^ at 100 µM concentration in the well‐established fluorogenic DiFMUP phosphatase assay [58] (Table 1, Table S1, Figure S4A). In brief, SHP2 was activated by a *bis*‐phosphotyrosyl peptide and was subsequently incubated with the fragments. Inhibition was quantified by the decrease in fluorescence intensity upon reduced SHP2 phosphatase activity. The SF library contained a diverse set of aryl SFs with a range of sizes and reactivities as described [56]. The SF warhead was chosen due to the absence of any cysteine residue in the targeted tunnel allosteric site and its latent reactivity towards lysine, tyrosine, and histidine side chains [59, 60], which held promise for the selective targeting of the intended tunnel allosteric binding site.

**TABLE 1:** Inhibition and covalent labeling of non‐biased sulfonyl fluoride (SF) fragments tested against full‐length SHP2 (SHP2^FL^).

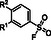					
Entry	R^1^	R^2^	% Inhibition @ 100 µM^a^	IC_50_, µM	Site(s) of covalent modification^b^
**SF1**	H	H	17% ± 6%	n.d.	n.d.
**SF2**	COOH	H	6% ± 0%	n.d.	n.d.
**SF3**	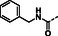	H	25% ± 2%	41 ± 11	Y66, Y81, K199, H426, Y511
**SF4**	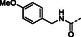	H	13% ± 2%	n.d.	n.d.
**SF5**	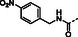	H	32% ± 3%	35 ± 6	Y66, Y81, K198, H426
**SF6**	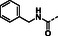	OMe	0%	n.d.	n.d.
**SF7**	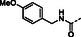	OMe	0%	n.d.	n.d.
**SF8**	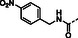	OMe	0%	n.d.	n.d.

*Note:* Values are mean ± S.D.

Abbreviation: n.d., not determined.

a DiFMUP phosphatase assay was used to determine % inhibition (at 100 µM inhibitor concentration) or inhibitor titration for IC_50_ determination against SHP2^FL^.

b Proteolytic digestion followed by MS/MS peptide fingerprinting was used to determinate the sites of covalent modification. A detailed description of the experiments is provided in the SI.

Phenylsulfonyl fluoride (**SF1**) and 3‐carboxyphenylsulfonyl fluoride (**SF2**) both provided weak inhibition (17% and 6% at 100 µM, respectively), in line with their low molecular weight [41] (Table 1). Growing **SF2** by acylating benzylamine (**SF3**), 4‐methoxybenzylamine (**SF4**) or 4‐nitrobenzylamine (**SF5**), increased the observed inhibition to 25%, 13% and 32% at 100 µM, respectively. Adding a *para*‐methoxy group to the phenyl SF moiety abolished the activity (**SF6**‐**SF8** showed no inhibition at 100 µM). The reported lower lysine and tyrosine reactivity of *para*‐methoxy substituted aryl SFs compared to matched pairs lacking this electron‐donating group that reduces the electrophilicity of the S(VI) center [55, 60] provides a possible explanation for the lower SHP2 inhibition by **SF6**‐**SF8** compared to **SF3**‐**SF5**.

We next determined the IC_50_ values for the most active SF fragments **SF3** (IC_50_ 41 ± 11 µM) and **SF5** (IC_50_ 35 ± 6 µM) (Figure S1) and subsequently investigated whether they label SHP2 covalently (Table 1). To this end, SHP2^FL^ was incubated with the respective fragments in 50‐fold molar excess for 1 h at 37°C. The intact protein was then analyzed for covalent modification by MS, where mass shifts corresponding to a multiple of the fragment molecular weight minus hydrogen fluoride indicated covalent labeling (Figures S4B and S5, Table S2).

**FIGURE 3:**
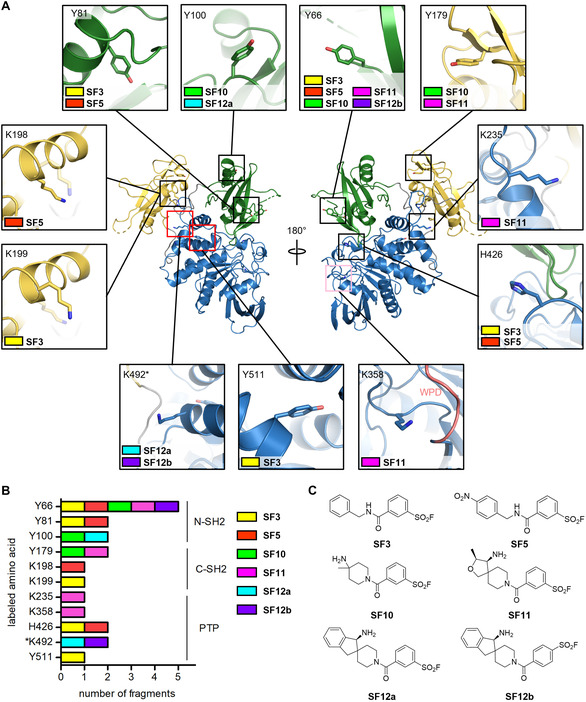
Overview of location and count of labeled amino acids of SHP2^FL^ by the tested aryl SF fragments. (A) Mapping of covalently modified amino acids on SHP2 (PDB ID 8T6G, domain‐ and binding site color coding as in Figure 1) illustrates distinct preferences for covalent modification by the tested SFs. Close‐up views of amino acids indicate the SFs that formed adducts with the respective residues, color‐coded as in panel B. K492, located in the targeted tunnel allosteric site, is marked with an asterisk (*). K358 is in proximity to the catalytic site´s conserved WPD loop, shown in salmon in the close‐up view. (B) Bar graph illustrating the number of SFs that formed covalent adducts detected by MS/MS with reactive amino acids, grouped by SHP2 domains (N‐terminal SH2, N‐SH2; C‐terminal SH2, C‐SH2, catalytic PTP domain). (C) Chemical structures of non‐biased SFs (**SF3** and **SF5**) and tunnel‐biased SFs (**SF10**‐**SF12**) that showed covalent binding to SHP2.

The sites of covalent modification were identified using MS/MS following tryptic digestion (Figures S6 and S7). Both fragments **SF3** and **SF5** showed multiple labeling of SHP2 at tyrosine, histidine and lysine residues (Table 1). An overlapping residue labeling pattern was observed. Primarily, surface‐exposed residues scattered over the N‐SH2 domain (Y66 and Y81), the C‐SH2 domain (K198, K199) and the PTP domain (H426 and Y511) were labeled (Figure 3), reflective of weak and transient interactions with the protein. In proximity to the tunnel allosteric binding site, only Y511 was labeled covalently by **SF3** but not by **SF5** (Figure 3) despite the presence of a number of potentially SF‐reactive amino acids in this pocket, including three lysine residues (K244, K260, K492, Figure 2A, Table S5). However, as these SF fragments were not designed specifically against SHP2, we did not expect selectivity toward any binding site, consistent with the multiple covalent modifications observed. Given the promiscuity of these fragments, we suspected that their inhibition of SHP2 was not a result of specific interactions at functionally relevant binding sites but rather stemmed from weak and transient noncovalent interactions and the altered protein surface properties upon multiple covalent modifications.

The matched fluorosulfate pairs of fragments **SF1**, **SF3**, and **SF5** were also tested for inhibition and covalent modification of SHP2^FL^. At 100 µM concentration, two fluorosulfate fragments showed slightly higher inhibitory activities compared to the corresponding SFs **SF1** and **SF3** (39% vs. 17% and 32% vs. 25%, respectively), while the fluorosulfate matched pair of **SF5** showed no significant inhibition of SHP2 (Table S1). No covalent labeling was observed by intact MS for any fluorosulfate fragment in line with the previously reported lower reactivity of fluorosulfates compared to SFs [59]. We postulate that noncovalent interactions between the non‐biased fragments and SHP2 are too short‐lived to enable covalent bond formation with the weakly electrophilic fluorosulfate warhead. The fact that matched fragment pairs lacking covalent reactivity towards SHP2 nevertheless inhibited the enzyme emphasizes noncovalent interactions as a contributing factor to the inhibitory activity of the tested fragments, albeit these are likely nonspecific.

Using this conventional SF fragment library, we demonstrated that amino acids beyond cysteine in full‐length SHP2 can be targeted covalently. However, multiple labeling suggests that fragment binding and the observed inhibitory effects are reactivity‐driven rather than the result of specific noncovalent interactions [42]. The covalently modified residues are located outside known druggable pockets of SHP2 and can therefore not be harnessed for TCI development (Figure 3).

To assess whether enhanced noncovalent interactions would lead to selective labeling of any residue in the functionally relevant tunnel allosteric site, especially lysine that reportedly has a lower intrinsic reactivity towards aryl SFs compared to tyrosine [55, 60], we turned to SHP2 tunnel site‐biased covalent fragments. These fragments combine the aryl SF warhead with noncovalent allosteric site‐targeted recognition elements. Given the relevance of noncovalent interactions for covalent target engagement [41], we hypothesized that the incorporation of SHP2 tunnel allosteric site pharmacophore features would guide the SF fragments toward the intended binding site and, through a sufficiently long lifetime of the noncovalent complex, would enable selective covalent engagement of weakly nucleophilic amino acids.

### 2.2 Allosteric Tunnel Site‐Biased Fragments

A conformationally constrained, primary amine is an invariant pharmacophore feature for allosteric SHP2 inhibitors [8], reflected in often spirocyclic, piperidine‐derived amino substituents (Figure 1B). Given the prevalence of these peculiar amine substructures in allosteric SHP2 inhibitors, we decided to use them as noncovalent recognition elements to guide SF fragments to the tunnel allosteric pocket, yielding a set of SHP2 tunnel allosteric site‐biased SF fragments. In analogy to non‐biased SF fragments, IC_50_ values against SHP2^FL^ were determined using the DiFMUP assay (Table 2, Figure S2). Sites of covalent modification were subsequently identified using tryptic digestion‐MS/MS (Table 2, Figure S4B and Figure S8–S11).

**TABLE 2:** Inhibition and sites of covalent modification by tunnel allosteric site‐biased sulfonyl fluoride (SF) fragments tested against full‐length SHP2 (SHP2^FL^). The reactive sulfonyl fluoride group is highlighted in dark red.

Entry	Structure	IC_50_, µM^a^	Sites of covalent modification^b^	*k*_inact_, min^−1^	*K*_I_, µM	*k*_inact_/*K*_I_, min^−1^ M^−1^
**SF10**	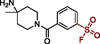	190 ± 40	Y66, Y100, Y179	n.d.	n.d.	n.d.
**SF11**	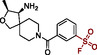	160 ± 20	Y66, Y179, K235, K358	n.d.	n.d.	n.d.
**SF12a**	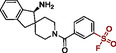	5.8 ± 1.0	K492, Y100	2 × 10^−3^	164	12
**SF12b**	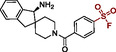	13 ± 5	K492, Y66	17 × 10^−3^	193	88

*Note*: Values are mean ± S.D. from independent duplicates.

Abbreviation: n.d., not determined.

a DiFMUP phosphatase assay was used for the inhibitor titration for IC_50_ determination against SHP2^FL^.

b Proteolytic digestion followed by MS/MS peptide fingerprinting was used to determine the sites of covalent modification. A detailed description of the experiments is provided in the SI.

The potency trend for the target‐biased SFs was overall consistent with published structure–activity relationship data for noncovalent allosteric SHP2 inhibitors [10, 61]. The 4‐methylpiperidin‐4‐amine‐derived SF **SF10** and (3*S*,4*S*)‐3‐methyl‐2‐oxa‐8‐azaspiro[4.5]decan‐4‐amine SF **SF11** showed moderate inhibitory potencies of 190 ± 40 and 160 ± 20 µM. 1,3‐Dihydrospiro[indene‐2,4^′^‐piperidin]‐1‐amine‐based SF **SF12a** displayed the highest inhibition of SHP2 with an IC_50_ of 5.8 ± 1.0 µM. Its *para*‐analog **SF12b** inhibited SHP2 with similar potency (IC_50_ 13 ± 5 µM). Surprisingly, the inhibitory potency of the studied SFs did not always correlate with their structural bias for the tunnel allosteric site. For example, the non‐biased SFs **SF3** and **SF5** showed similar or higher inhibition compared to the target‐biased SFs **SF10** and **SF11**. Different half‐lives in the buffer medium may also contribute to the observed activity differences. The more active fragments **SF3**, **SF5**, **SF12a**, and **SF12b** showed half‐lives of several hours in buffer (Table S3 and ref. [56]), while SHP2‐biased fragments **SF10** and **SF11** were more prone to hydrolysis (Table S3).

Only **SF12a** and **SF12b** displayed superior inhibitory potency compared to the non‐biased SFs. This suggests that the molecular size strongly influences the strength of interactions between the protein and the SFs. However, it is unclear whether the inhibitory effect of **SF10** and **SF11** resulted from noncovalent binding to the tunnel allosteric site or was a nonspecific effect as suspected of **SF3** and **SF5**. The low inhibitory potency of the target‐biased SFs compared to drug‐like allosteric SHP2 inhibitors can be explained by missing pharmacophore features required in addition to the conformationally constrained amine for targeting the tunnel allosteric site, namely an *N*‐heterocyclic core and a lipophilic substituent that forms a cation–π interaction with Arg111 [8, 57].

Despite only minor differences in inhibitory activity, distinct sets of amino acids were covalently modified by target‐biased versus non‐biased SFs (Figure 3). **SF10** labeled tyrosine residues Y66, Y100, and Y179. While Y66 was also labeled by non‐biased SFs, Y100 and Y179 were only reactive toward target‐biased SFs. Y66 and Y100 are located in the N‐SH2 domain, at 37 and 35 Å distance from the targeted tunnel binding site. These measurements refer to the spatial separation between the nucleophilic side chain atom and the piperidine nitrogen of the respective co‐crystallized allosteric SHP2 inhibitor, in this case **SHP099** (PDB ID 5EHR [8]). Y179 in the C‐SH2 domain is located closer to the tunnel site (19 Å in PDB ID 5EHR [8]). Like **SF10**, **SF11** formed covalent adducts with Y66 and Y179. In addition, K235 and K358 in the PTP domain were labeled. K235 is 16 Å away from the tunnel binding site (PDB ID 7JVM [9]), whereas K358 is adjacent to the conserved WPD loop that controls the catalytic activity of SHP2 [15] (Figure 3A). Interestingly, Day and colleagues recently reported a fragment hit binding to a cryptic pocket under the WPD loop [62], substantiating the presence of a low‐affinity secondary site in this region of the PTP domain. The largest biased SFs, **SF12a** and **SF12b**, showed a remarkable specificity: Apart from surface‐exposed Y100 in case of **SF12a** and Y66 in case of **SF12b**, only a single lysine residue, K492, in the hydrophobic part of the targeted allosteric binding tunnel was covalently modified. The high‐affinity noncovalent inhibitor **SHP099** [7, 8], known to bind to the targeted tunnel site of SHP2, reduced covalent modification of the intact protein by **SF12a**, confirming that **SF12a** binds to the same tunnel site (Figure S12). K492 was not labeled by the other assayed SFs devoid of the structurally complex noncovalent amine recognition element of **SF12a** and **SF12b**. Enhanced noncovalent interactions that precede covalent bond formation and that determine the lifetime of the noncovalent complex [21] seem to facilitate covalent engagement of this otherwise unreactive residue. We presume that the superior inhibitory potency of **SF12a** and **SF12b** compared to the smaller SFs is in part attributable to covalent binding to K492, that is located inside the tunnel‐like allosteric site addressed by clinical‐stage SHP2 inhibitors (shown in Figure 1B). **SF12a** and **SF12b** likely inhibit SHP2 through a similar mechanism as these drug‐like noncovalent inhibitors, namely through the stabilization of the autoinhibited conformation. While Y511, labeled covalently by **SF3**, is also in proximity to the tunnel binding site (Figure 3A), its hydroxyl group appears to be less accessible. This may result in weaker labeling and might therefore not have such a strong effect on potency as the covalent engagement of K492. The covalent modification of amino acids that are located outside functionally relevant binding sites, such as Y66, had a weaker inhibitory effect. Thus, the observed inhibition upon labeling of these surface‐exposed residues might be attributable to altered surface properties that induce protein aggregation or affect protein stability rather than to a specific effect on phosphatase activity.

As both the rate of covalent bond formation and noncovalent interactions between the inhibitor and the protein determine its potency, reflected in the IC_50_ [21], we determined the noncovalent affinity (*K*_I_) and the inactivation rate constant (*k*_inact_) of **SF12a** and **SF12b** as well as the ratio *k*_inact_/*K*_I_ (Table 2, Figure S13). **SF12a** and **SF12b** displayed *k*_inact_/*K*_I_ values of 12 and 88 min^−1^ M^−1^. While both compounds had comparable noncovalent affinities (*K*_I_ = 164 and 193 µM for **SF12a** and **SF12b**, respectively), **SF12b** exhibited a slightly higher rate of covalent modification of SHP2 (*k*_inact_ 17 × 10^−3^ min^−1^) compared to **SF12a** (2 × 10^−3^ min^−1^). While these *k*_inact_ values are low compared to cysteine‐reactive warheads, they are in the same range as previously reported, similarly sized SFs [60, 63].

To further investigate the interplay of covalent and noncovalent interactions in the context of the target‐biased fragments, we designed structurally similar analogs of the most active SF fragments (Table 3). The fluorosulfate matched pairs of **SF12a** and **SF12b**, **OSF12a** and **OSF12b**, were tested to determine whether the improved noncovalent interactions would activate the less electrophilic fluorosulfate warhead for covalent bond formation. **OSF12a** and **OSF12b** did not show covalent labeling of SHP2 in line with the non‐biased fluorosulfate fragments. However, in contrast to the non‐biased fragments, they showed very weak inhibition of SHP2. Likewise, analogs of **SF12a** and **SF12b** devoid of covalent warheads showed no (**13a** and **14**) or strongly reduced (**13b**) inhibition of SHP2 despite their similar size and the presence of the spiroamine tunnel‐targeting substructure (Figure S3). This underscores the contribution of covalent engagement of K492 to the inhibitory potency of **SF12a** and **SF12b** in addition to enhanced noncovalent interactions. Also, it challenges the notion that the enhanced inhibition of SHP2 by **SF12a** and **SF12b** could solely result from differences in molecular weight [41]. Conversely, we observed that the noncovalent recognition element of **SF12a** and **SF12b** was required for covalent bond formation with K492 in the targeted tunnel allosteric site, which in turn enhanced phosphatase inhibition. Given the slow covalent reaction kinetics of **SF12a** and **SF12b**, their noncovalent interactions must be strong enough to retain the fragments within proximity to K492 for a sufficiently long time to enable covalent binding.

**TABLE 3:** Inhibitory potencies of spiroamine‐derived SHP2 tunnel allosteric site‐biased aryl fluorosulfate‐ (OSF) and noncovalent matched pairs of the most potent biased fragments tested against full‐length SHP2 (SHP2^FL^). The reactive fluorosulfate group is highlighted in dark red.

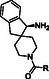			
Entry	R	IC_50_, µM^a^	Sites of covalent modification^b^
**OSF12a**	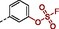	11% ± 9% inhibition @ 100 µM	‐‐
**OSF12b**	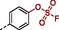	7% ± 5% inhibition @ 100 µM	‐‐
**13a**	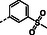	>1000	n.a.
**13b**	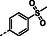	93 ± 36	n.a.
**14**	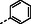	>1000	n.a.

*Note*: Values are mean ± S.D.

Abbreviations: ‐‐, no labeling observed; n.a., not applicable.

a DiFMUP phosphatase assay was used for IC_50_ determination against SHP2^FL^.

b Proteolytic digest followed by MS/MS peptide fingerprinting was used determination of the sites of covalent modification. A detailed description of the experiments is provided in the SI.

### 2.3 Binding Modes of the Most Potent Target‐Biased SFs

**FIGURE 4:**
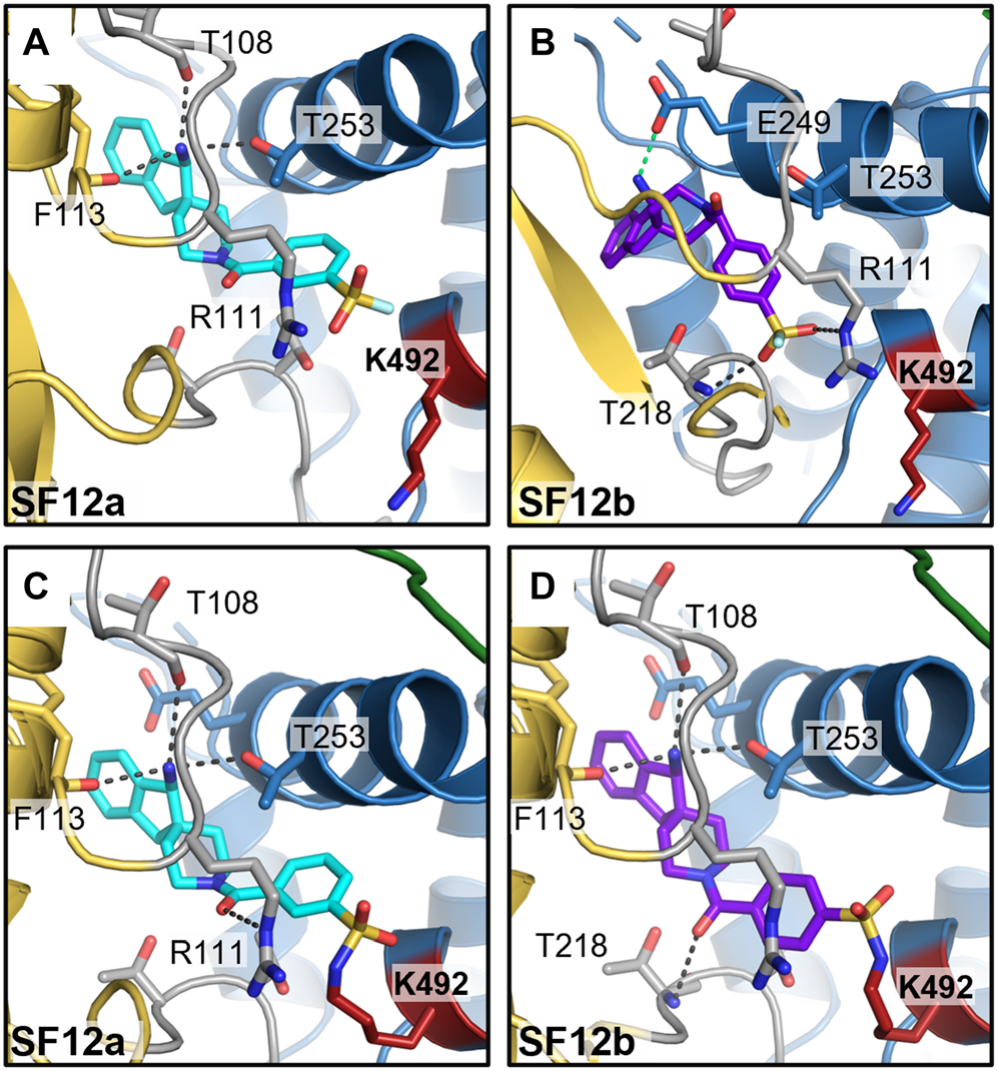
Docking poses of the most active SHP2 tunnel allosteric site‐biased aryl SFs: (A,B) Noncovalent docking poses of **SF12a** (A, ligand shown as cyan sticks) and **SF12b** (B, ligand shown as purple sticks). H‐bonds are represented by gray dashed lines; salt bridge shown as green dashed line in (B). The targeted amino acid K492 is highlighted in red. Distances between the S(VI) center and ε‐NH_2_ nitrogen of K492 are 8.2 and 9.5 Å, respectively. (C,D) Covalent docking poses showing adduct formation between K492 (red) and the SF warheads of **SF12a** and **SF12b** that was observed in MS/MS experiments. SHP2 domain color coding as in Figure 2.

We investigated possible binding modes of the target‐biased SFs by docking and the analysis of X‐ray co‐crystal structures. All biased SFs can be accommodated and form favorable interactions in the tunnel allosteric site. In noncovalent docking poses, the primary amines of **SF12a** (Figure 4A) and of the less active SFs **SF10** and **SF11** (Figure S14A,B) can engage in H‐bonds with T108, F113, and T253 amongst others, similar to the corresponding drug‐like allosteric SHP2 inhibitors featuring the respective amine substructures [8, 9, 10]. In contrast, the benzylic amine of **SF12b** forms a salt bridge with E249 (Figure 4B). The aryl SF moieties are oriented toward K492. Additional H‐bonds are predicted between the SF oxygens and the R111 side chain as well as the backbone NH of T218. The largest biased fragments **SF12a** and **SF12b** show improved shape complementarity to the tunnel binding site, providing a possible explanation for their superior potency compared to the smaller SFs. Covalent docking suggests a conformational change of the K492 side chain towards the S(VI) center of **SF12a** and **SF12b** (Figure 4C,D). We also noticed the proximity between the SF warheads of **SF12a** and **SF12b** and the guanidinium group of R111 that might accelerate covalent bond formation as proposed earlier for S(VI) warheads targeting tyrosines [64, 65]. Upon covalent bond formation, **SF12a** is predicted to retain its binding pose, while **SF12b** shifts closer to K492, enabling the formation of the three H‐bonds observed for **SF12a** and for drug‐like noncovalent allosteric SHP2 inhibitors such as **GDC‐1971** [10] (Figure S14D). We attempted co‐crystallization of **SF12a** with SHP2^FL^. The resulting co‐crystal structure confirmed the binding of the fragment to the targeted tunnel allosteric site. However, although covalent labeling has been proven by MS and enzymatic digestion, instead of **SF12a**, its aryl sulfonate hydrolysis product was generated during the prolonged incubation under basic crystallization conditions (7 days, pH 8.5) and was observed in the obtained X‐ray co‐crystal structure (Figure S14C).

## 3 Conclusion

We compared sets of small, generic non‐biased aryl SF fragments and target‐biased aryl SF fragments designed to bind to the tunnel allosteric pocket of SHP2 in terms of SHP2 inhibition and covalent modification of the enzyme. Non‐biased SFs promiscuously labeled surface‐exposed tyrosine, histidine, and lysine residues, resulting in weak phosphatase inhibition. Target‐biased SF fragments that feature amine moieties optimized for noncovalent binding to the tunnel allosteric site of SHP2 showed distinct labeling preferences, albeit nonspecific covalent binding was also observed for small target‐biased SFs. Also, the incorporation of tunnel allosteric site‐targeting pharmacophores did not necessarily translate into stronger SHP2 inhibition. Only the biased SFs with the most complex noncovalent recognition element, **SF12a** and **SF12b**, displayed increased inhibitory potencies over non‐biased SFs in the low µM range and showed covalent binding to the tunnel allosteric pocket through K492. K492 was not labeled by any other tested SF, nor by a recently reported aryl SF‐containing small molecule allosteric SHP2 inhibitor [11]. Covalent engagement of K492 enhanced the inhibitory potency of the biased SFs since noncovalent analogs as well as less reactive fluorosulfate matched pairs showed no or strongly reduced inhibition of SHP2. Nevertheless, even the most selective target‐biased SFs showed off‐target labeling of one tyrosine residue remote from the targeted allosteric pocket.

In this study, we demonstrate that aryl SFs are suitable warheads for covalent allosteric inhibition of SHP2 through engagement of K492 in the tunnel binding site. The screening of our target‐biased‐ and non‐biased SF fragment sets presents the first systematic approach towards charting covalent interaction sites in full‐length SHP2 with emphasis on the tunnel allosteric site. The findings reported herein can serve as a starting point for the development of K492‐targeting allosteric SHP2 TCIs that might have the potential to overcome reduced inhibitor efficacy in the presence of activating mutations.

More broadly, our study illustrates how noncovalent recognition influences covalent fragment binding. Consequently, conventional SF fragments that form weak and transient interactions failed to identify ligandable residues in the relevant tunnel allosteric site of SHP2. By exploiting allosteric SHP2 inhibitor pharmacophore elements, we identified a reactive nucleophilic acid in the targeted tunnel allosteric site that would not have emerged from screening the non‐biased covalent fragment library in a classical covalent mapping setup. Our approach, based on target‐biased covalent fragments that feature noncovalent site‐directed substructures, can expedite TCI development without requiring a priori knowledge of ligandable nucleophilic residues. Overall, for pharmacologically validated targets, biased covalent fragments can offer advantages over generic covalent fragments missing specific interactions, and over a ligand‐first approach that relies on predicted amino acid reactivities.

## Supporting Information

Additional supporting information can be found online in the Supporting Information section.

## Author Contributions

**Nina‐Louisa Efrém**: conceptualization, methodology, investigation, validation, formal analysis, writing – original draft, writing – review & editing, data curation, visualization. **Noémi Csorba**: investigation, validation, formal analysis, writing – review and editing, data curation. **Machoud Amoussa**: conceptualization, investigation, writing – review & editing, formal analysis. **Peter Abranyi‐Balogh**: supervision, validation, writing – review & editing. **Ziqiong Guo**: conceptualization, validation, formal analysis, investigation. **László Petri**: investigation, formal analysis. **Feng Bo**: investigation, methodology, formal analysis, data curation. **Yvette Roske**: methodology, investigation, validation, formal analysis, visualization, writing – review and editing. **Vincenzo Di Lorenzo**: investigation, formal analysis. **Tibor Viktor Szalai**: formal analysis, investigation. **Levente Mihalovits**: investigation, visualization. **József Simon**: formal analysis, visualization. **Jia Li**: conceptualization, supervision, funding acquisition, resources, writing – review and editing. **Oliver Daumke**: data curation, investigation, formal analysis, writing ‐ review and editing, supervision, resources. **György Keserü**: conceptualization, supervision, funding acquisition, project administration, resources. **Marc Nazaré**: conceptualization, writing – review and editing, writing – original draft, visualization, supervision, funding acquisition, investigation, formal analysis, validation, project administration, resources.

## Funding

This study was supported by the European Union's Horizon 2020 research and innovation program, Marie Skłodowska Curie grant agreement no. 956314 ALLODD, by the National Research Development and Innovation Office of Hungary (PharmaLab RRF‐2.3.1‐21‐2022‐00015, NKKP Starting 152137), and by a János Bolyai Research Scholarship of the Hungarian Academy of Sciences.

## Conflicts of Interest

The authors declare no conflicts of interest.

## Supporting information

Supplementary Material

## Data Availability

The data that support the findings of this study are available from the corresponding authors upon reasonable request.

## References

[1] B. G. Neel , H. Gu , and L. Pao , “The ’Shp’ing News: SH2 Domain‐Containing Tyrosine Phosphatases in Cell Signaling,” Trends in Biochemical Sciences 28 (2003): 284–293. 12826400 10.1016/S0968-0004(03)00091-4

[2] K. S. Grossmann , M. Rosário , C. Birchmeier , and W. Birchmeier , “The Tyrosine Phosphatase Shp2 in Development and Cancer,” Advances in Cancer Research 106 (2010): 53–89. 20399956 10.1016/S0065-230X(10)06002-1

[3] N. M. Sodir , G. Pathria , J. I. Adamkewicz , et al., “SHP2: A Pleiotropic Target at the Interface of Cancer and Its Microenvironment,” Cancer Discovery 13 (2023): 2339–2355. 37682219 10.1158/2159-8290.CD-23-0383 PMC10618746

[4] J. Bendell , S. Ulahannan , M. Koczywas , et al., “Intermittent Dosing of RMC‐4630, a Potent, Selective Inhibitor of SHP2, Combined with the MEK Inhibitor Cobimetinib, in a Phase 1b/2 Clinical Trial for Advanced Solid Tumors with Activating Mutations of RAS Signaling,” European Journal of Cancer 138 (2020): S8–S9.

[5] M. V. Negrao , P. A. Cassier , B. Solomon , et al., “MA06.03 KontRASt‐01: Preliminary Safety and Efficacy of JDQ443 + TNO155 in Patients with Advanced, KRAS G12C‐Mutated Solid Tumors,” Journal of Thoracic Oncology 18 (2023): S117–S118.

[6] M. Fodor , E. Price , P. Wang , et al., “Dual Allosteric Inhibition of SHP2 Phosphatase,” ACS Chemical Biology 13 (2018): 647–656. 29304282 10.1021/acschembio.7b00980

[7] Y. N. Chen , M. J. LaMarche , H. M. Chan , et al., “Allosteric Inhibition of SHP2 Phosphatase Inhibits Cancers Driven by Receptor Tyrosine Kinases,” Nature 535 (2016): 148–152. 27362227 10.1038/nature18621

[8] J. Garcia Fortanet , C. H. Chen , Y. N. Chen , et al., “Allosteric Inhibition of SHP2: Identification of a Potent, Selective, and Orally Efficacious Phosphatase Inhibitor,” Journal of Medicinal Chemistry 59 (2016): 7773–7782. 27347692 10.1021/acs.jmedchem.6b00680

[9] M. J. LaMarche , M. Acker , A. Argintaru , et al., “Identification of TNO155, an Allosteric SHP2 Inhibitor for the Treatment of Cancer,” Journal of Medicinal Chemistry 63 (2020): 13578–13594. 32910655 10.1021/acs.jmedchem.0c01170

[10] A. M. Taylor , B. R. Williams , F. Giordanetto , et al., “Identification of GDC‐1971 (RLY‐1971), a SHP2 Inhibitor Designed for the Treatment of Solid Tumors,” Journal of Medicinal Chemistry 66 (2023): 13384–13399. 37774359 10.1021/acs.jmedchem.3c00483

[11] M. Zhang , S. Wu , M. Liu , et al., “Structure‐Guided Expansion Strategy Unveils Potent Allosteric SHP2 Inhibitors with Synergistic Efficacy against AML through MCL‐1 Co‐Targeting,” European Journal of Medicinal Chemistry 298 (2025): 117988. 40730062 10.1016/j.ejmech.2025.117988

[12] W. Liang , A. D. Krabill , K. S. Gallagher , et al., “Natural Product‐Inspired Molecules for Covalent Inhibition of SHP2 Tyrosine Phosphatase,” Tetrahedron 156 (2024): 133918. 38618612 10.1016/j.tet.2024.133918 PMC11008911

[13] B. Marsh‐Armstrong , J. M. Fajnzylber , S. Korntner , B. A. Plaman , and A. C. Bishop , “The Allosteric Site on SHP2's Protein Tyrosine Phosphatase Domain Is Targetable with Druglike Small Molecules,” ACS Omega 3 (2018): 15763–15770. 30533581 10.1021/acsomega.8b02200 PMC6275946

[14] P. Hof , S. Pluskey , S. Dhe‐Paganon , M. J. Eck , and S. E. Shoelson , “Crystal Structure of the Tyrosine Phosphatase SHP‐2,” Cell 92 (1998): 441–450. 9491886 10.1016/s0092-8674(00)80938-1

[15] D. Barford and B. G. Neel , “Revealing Mechanisms for SH2 Domain Mediated Regulation of the Protein Tyrosine Phosphatase SHP‐2,” Structure 6 (1998): 249–254. 9551546 10.1016/s0969-2126(98)00027-6

[16] M. Tartaglia , E. L. Mehler , R. Goldberg , et al., “Mutations in PTPN11, Encoding the Protein Tyrosine Phosphatase SHP‐2, cause Noonan Syndrome,” Nature Genetics 29 (2001): 465–468. 11704759 10.1038/ng772

[17] M. Tartaglia , C. M. Niemeyer , A. Fragale , et al., “Somatic Mutations in PTPN11 in Juvenile Myelomonocytic Leukemia, Myelodysplastic Syndromes and Acute Myeloid Leukemia,” Nature Genetics 34 (2003): 148–150. 12717436 10.1038/ng1156

[18] J. R. LaRochelle , M. Fodor , X. Xu , et al., “Structural and Functional Consequences of Three Cancer‐Associated Mutations of the Oncogenic Phosphatase SHP2,” Biochemistry 55 (2016): 2269–2277. 27030275 10.1021/acs.biochem.5b01287 PMC4900891

[19] R. A. P. Padua , Y. Sun , I. Marko , et al., “Mechanism of Activating Mutations and Allosteric Drug Inhibition of the Phosphatase SHP2,” Nature Communications 9 (2018): 4507. 10.1038/s41467-018-06814-w PMC6207724 30375376

[20] J. R. LaRochelle , M. Fodor , V. Vemulapalli , et al., “Structural Reorganization of SHP2 by Oncogenic Mutations and Implications for Oncoprotein Resistance to Allosteric Inhibition,” Nature Communications 9 (2018): 4508. 10.1038/s41467-018-06823-9 PMC6207684 30375388

[21] J. Singh , R. C. Petter , T. A. Baillie , and A. Whitty , “The Resurgence of Covalent Drugs,” Nature Reviews Drug Discovery 10 (2011): 307–317. 21455239 10.1038/nrd3410

[22] T. A. Carter , L. M. Wodicka , N. P. Shah , et al., “Inhibition of Drug‐Resistant Mutants of ABL, KIT, and EGF Receptor Kinases,” Proceedings of the National Academy of Sciences 102 (2005): 11011–11016. 10.1073/pnas.0504952102 PMC1180625 16046538

[23] R. Nussinov and C. J. Tsai , “The Design of Covalent Allosteric Drugs,” Annual Review of Pharmacology and Toxicology 55 (2015): 249–267. 10.1146/annurev-pharmtox-010814-124401 25149918

[24] H. Tao , B. Yang , A. Farhangian , et al., “Covalent‐Allosteric Inhibitors: Do We Get the Best of Both Worlds?,” Journal of Medicinal Chemistry 68 (2025): 4040–4052. 39937154 10.1021/acs.jmedchem.4c02760 PMC12207613

[25] H. Kim , Y. S. Hwang , M. Kim , and S. B. Park , “Recent Advances in the Development of Covalent Inhibitors,” RSC Medicinal Chemistry 12 (2021): 1037–1045. 34355176 10.1039/d1md00068c PMC8292994

[26] D. E. Heppner , B. C. Ogboo , D. A. Urul , et al., “Demystifying Functional Parameters for Irreversible Enzyme Inhibitors,” Journal of Medicinal Chemistry 67 (2024): 14693–14696. 39115869 10.1021/acs.jmedchem.4c01721 PMC12057623

[27] G. Akcay , M. A. Belmonte , B. Aquila , et al., “Inhibition of Mcl‐1 through Covalent Modification of a Noncatalytic Lysine Side Chain,” Nature Chemical Biology 12 (2016): 931–936. 27595327 10.1038/nchembio.2174

[28] Q. Zhao , X. Ouyang , X. Wan , et al., “Broad‐Spectrum Kinase Profiling in Live Cells with Lysine‐Targeted Sulfonyl Fluoride Probes,” Journal of the American Chemical Society 139 (2017): 680–685. 28051857 10.1021/jacs.6b08536 PMC5858558

[29] L. A. Crawford and E. Weerapana , “A Tyrosine‐Reactive Irreversible Inhibitor for Glutathione S‐Transferase Pi (GSTP1),” Molecular Omics 12 (2016): 1768–1771. 10.1039/c6mb00250a PMC4879005 27113843

[30] Y. Seki , T. Ishiyama , D. Sasaki , et al., “Transition Metal‐Free Tryptophan‐Selective Bioconjugation of Proteins,” Journal of the American Chemical Society 138 (2016): 10798–10801. 27534812 10.1021/jacs.6b06692

[31] K. W. Decoene , K. Unal , A. Staes , et al., “Triazolinedione Protein Modification: From an Overlooked Off‐Target Effect to a Tryptophan‐Based Bioconjugation Strategy,” Chemical Sciences 13 (2022): 5390–5397. 10.1039/d1sc06942j PMC9093138 35655564

[32] K. Nakane , S. Sato , T. Niwa , et al., “Proximity Histidine Labeling by Umpolung Strategy Using Singlet Oxygen,” Journal of the American Chemical Society 143 (2021): 7726–7731. 33904715 10.1021/jacs.1c01626

[33] K. Otrubova , S. Chatterjee , S. Ghimire , B. F. Cravatt , and D. L. Boger , “N‐Acyl Pyrazoles: Effective and Tunable Inhibitors of Serine Hydrolases,” Bioorganic & Medicinal Chemistry 27 (2019): 1693–1703. 30879861 10.1016/j.bmc.2019.03.020 PMC6474344

[34] J. Yang , L. A. R. Carvalho , S. Ji , S. Chen , R. Moreira , and S. H. L. Verhelst , “4‐Oxo‐Beta‐Lactams as Novel Inhibitors for Rhomboid Proteases,” Chembiochem : A European Journal of Chemical Biology 24 (2023): e202300418. 37671979 10.1002/cbic.202300418

[35] D. B. Diaz and A. K. Yudin , “The Versatility of Boron in Biological Target Engagement,” Nature Chemistry 9 (2017): 731–742. 10.1038/nchem.2814 28754930

[36] A. Gonzalez‐Valero , A. G. Reeves , A. C. S. Page , et al., “An Activity‐Based Oxaziridine Platform for Identifying and Developing Covalent Ligands for Functional Allosteric Methionine Sites: Redox‐Dependent Inhibition of Cyclin‐Dependent Kinase 4,” Journal of the American Chemical Society 144 (2022): 22890–22901. 36484997 10.1021/jacs.2c04039 PMC10124963

[37] S. Li , P. Zhang , F. Xu , et al., “Ynamide Electrophile for the Profiling of Ligandable Carboxyl Residues in Live Cells and the Development of New Covalent Inhibitors,” Journal of Medicinal Chemistry 65 (2022): 10408–10418. 35880853 10.1021/acs.jmedchem.2c00272

[38] Y. D. Petri , C. S. Gutierrez , and R. T. Raines , “Chemoselective Caging of Carboxyl Groups for On‐Demand Protein Activation with Small Molecules,” Angewandte Chemie International Edition 62 (2023): e202215614. 36964973 10.1002/anie.202215614 PMC10243506

[39] N. Csorba , P. Abranyi‐Balogh , and G. M. Keseru , “Covalent Fragment Approaches Targeting Non‐Cysteine Residues,” Trends in Pharmacological Sciences 44 (2023): 802–816. 37770315 10.1016/j.tips.2023.08.014

[40] L. M. McGregor , M. L. Jenkins , C. Kerwin , J. E. Burke , and K. M. Shokat , “Expanding the Scope of Electrophiles Capable of Targeting K‐Ras Oncogenes,” Biochemistry 56 (2017): 3178–3183. 28621541 10.1021/acs.biochem.7b00271 PMC5665167

[41] L. Petri , R. Gabizon , G. G. Ferenczy , et al., “Size‐Dependent Target Engagement of Covalent Probes,” Journal of Medicinal Chemistry 68 (2025): 6616–6632. 40099438 10.1021/acs.jmedchem.5c00017 PMC11956015

[42] S. E. Dalton and S. Campos , “Covalent Small Molecules as Enabling Platforms for Drug Discovery,” Chembiochem: A European Journal of Chemical Biology 21 (2020): 1080–1100. 31833626 10.1002/cbic.201900674

[43] P. Punthasee , A. R. Laciak , A. H. Cummings , et al., “Covalent Allosteric Inactivation of Protein Tyrosine Phosphatase 1B (PTP1B) by an Inhibitor‐Electrophile Conjugate,” Biochemistry 56 (2017): 2051–2060. 28345882 10.1021/acs.biochem.7b00151

[44] A. Keeley , L. Petri , P. Abranyi‐Balogh , and G. M. Keseru , “Covalent Fragment Libraries in Drug Discovery,” Drug Discovery Today 25 (2020): 983–996. 32298798 10.1016/j.drudis.2020.03.016

[45] E. Resnick , A. Bradley , J. Gan , et al., “Rapid Covalent‐Probe Discovery by Electrophile‐Fragment Screening,” Journal of the American Chemical Society 141 (2019): 8951–8968. 31060360 10.1021/jacs.9b02822 PMC6556873

[46] R. M. Miller , V. O. Paavilainen , S. Krishnan , I. M. Serafimova , and J. Taunton , “Electrophilic Fragment‐Based Design of Reversible Covalent Kinase Inhibitors,” Journal of the American Chemical Society 135 (2013): 5298–5301. 23540679 10.1021/ja401221b PMC3665406

[47] S. G. Kathman , Z. Xu , and A. V. Statsyuk , “A Fragment‐Based Method to Discover Irreversible Covalent Inhibitors of Cysteine Proteases,” Journal of Medicinal Chemistry 57 (2014): 4969–4974. 24870364 10.1021/jm500345q PMC4113264

[48] K. M. Backus , B. E. Correia , K. M. Lum , et al., “Proteome‐Wide Covalent Ligand Discovery in Native Biological Systems,” Nature 534 (2016): 570–574. 27309814 10.1038/nature18002 PMC4919207

[49] M. E. Abbasov , M. E. Kavanagh , T. A. Ichu , et al., “A Proteome‐Wide Atlas of Lysine‐Reactive Chemistry,” Nature Chemistry 13 (2021): 1081–1092. 10.1038/s41557-021-00765-4 PMC8952960 34504315

[50] S. M. Hacker , K. M. Backus , M. R. Lazear , S. Forli , B. E. Correia , and B. F. Cravatt , “Global Profiling of Lysine Reactivity and Ligandability in the Human Proteome,” Nature Chemistry 9 (2017): 1181–1190. 10.1038/nchem.2826 PMC5726523 29168484

[51] C. M. Chio , C. S. Lim , and A. C. Bishop , “Targeting a Cryptic Allosteric Site for Selective Inhibition of the Oncogenic Protein Tyrosine Phosphatase Shp2,” Biochemistry 54 (2015): 497–504. 25519989 10.1021/bi5013595 PMC4303306

[52] S. H. Hong , S. Y. Xi , A. C. Johns , et al., “Mapping the Chemical Space of Active‐Site Targeted Covalent Ligands for Protein Tyrosine Phosphatases,” Chembiochem: A European Journal of Chemical Biology 24 (2023): e202200706. 36893077 10.1002/cbic.202200706 PMC10192133

[53] F. H. G. Ter Brake , S. van Luttikhuizen , T. van der Wel , et al., “Previously Published Phosphatase Probes Have Limited Utility Due to Their Unspecific Reactivity,” Chembiochem : A European Journal of Chemical Biology 25 (2024): e202400333. 39229773 10.1002/cbic.202400333

[54] A. Narayanan and L. H. Jones , “Sulfonyl Fluorides as Privileged Warheads in Chemical Biology,” Chemical Science 6 (2015): 2650–2659. 28706662 10.1039/c5sc00408j PMC5489032

[55] H. Mukherjee , J. Debreczeni , J. Breed , et al., “A Study of the Reactivity of S(VI)‐F Containing Warheads with Nucleophilic Amino‐Acid Side Chains under Physiological Conditions,” Organic & Biomolecular Chemistry 15 (2017): 9685–9695. 29119993 10.1039/c7ob02028g

[56] L. Petri , P. Abranyi‐Balogh , N. Csorba , et al., “Activation‐Free Sulfonyl Fluoride Probes for Fragment Screening,” Molecules 28 (2023): 3042. 37049805 10.3390/molecules28073042 PMC10096327

[57] A. Petrocchi and A. Ciammaichella , “A Patent Review of SHP2 Allosteric Inhibitors (2018‐Present),” Expert Opinion on Therapeutic Patents 34 (2024): 383–396. 38842843 10.1080/13543776.2024.2365410

[58] S. Welte , K. H. Baringhaus , W. Schmider , G. Muller , S. Petry , and N. Tennagels , “6,8‐Difluoro‐4‐Methylumbiliferyl Phosphate: A Fluorogenic Substrate for Protein Tyrosine Phosphatases,” Analytical Biochemistry 338 (2005): 32–38. 15707933 10.1016/j.ab.2004.11.047

[59] J. Dong , L. Krasnova , M. G. Finn , and K. B. Sharpless , “Sulfur(VI) Fluoride Exchange (SuFEx): Another Good Reaction for Click Chemistry,” Angewandte Chemie International Edition 53 (2014): 9430–9448. 25112519 10.1002/anie.201309399

[60] K. E. Gilbert , A. Vuorinen , A. Aatkar , et al., “Profiling Sulfur(VI) Fluorides as Reactive Functionalities for Chemical Biology Tools and Expansion of the Ligandable Proteome,” ACS Chemical Biology 18 (2023): 285–295. 36649130 10.1021/acschembio.2c00633 PMC9942091

[61] E. Torrente , V. Fodale , A. Ciammaichella , et al., “Discovery of a Novel Series of Imidazopyrazine Derivatives as Potent SHP2 Allosteric Inhibitors,” ACS Medicinal Chemistry Letters 14 (2023): 156–162. 36793438 10.1021/acsmedchemlett.2c00454 PMC9923835

[62] J. E. H. Day , V. Berdini , J. Castro , et al., “Fragment‐Based Discovery of Allosteric Inhibitors of SH2 Domain‐Containing Protein Tyrosine Phosphatase‐2 (SHP2),” Journal of Medicinal Chemistry 67 (2024): 4655–4675. 38462716 10.1021/acs.jmedchem.3c02118

[63] A. Aatkar , A. Vuorinen , and O. E. Longfield , “Efficient Ligand Discovery Using Sulfur(VI) Fluoride Reactive Fragments,” ACS Chemical Biology 18 (2023): 1926–1937. 37084287 10.1021/acschembio.3c00034 PMC10510102

[64] M. Teng , S. B. Ficarro , H. Yoon , et al., “Rationally Designed Covalent BCL6 Inhibitor That Targets a Tyrosine Residue in the Homodimer Interface,” ACS Medicinal Chemistry Letters 11 (2020): 1269–1273. 32551010 10.1021/acsmedchemlett.0c00111 PMC7294706

[65] L. Cao , B. Yu , P. C. Klauser , P. Zhang , S. Li , and L. Wang , “Arginine Accelerates Sulfur Fluoride Exchange and Phosphorus Fluoride Exchange Reactions between Proteins,” Angewandte Chemie International Edition 63 (2024): e202412843. 39113386 10.1002/anie.202412843 PMC11560669

