# Supplementary material for: Mapping the SHP2 Allosteric Pocket With Target‐Biased Covalent Fragments
Source: Chembiochem. 2026 Apr 24;27(8):e70310. doi: 10.1002/cbic.70310 (PMC13109685; doi:10.1002/cbic.70310)
Supplement: Supplementary file 1 — Supplementary Material [file CBIC-27-e70310-s001.pdf]

Supporting Information

Mapping the SHP2 allosteric pocket with target-biased covalent fragments

Nina-Louisa Efrém,[a,b] Noémi Csorba,[c,d] Machoud Amoussa,[a,e] Péter Ábrányi-Balogh,[c,d] Ziqiong Guo,[a,e] László Petri,[c] Feng Bo,[f] Vincenzo Di Lorenzo,[c,d] Yvette Roske,[g] Tibor Viktor Szalai,[c] Levente Mihalovits,[c] József Simon,[c] Jia Li,[f] Oliver Daumke,[g] György M. Keserű,*[c,d] Marc Nazaré*[a,h]

[a] N.-L. Efrém, M. Amoussa, Z. Guo, M. Nazaré
Leibniz-Forschungsinstitut für Molekulare Pharmakologie (FMP), Medicinal Chemistry
Campus Berlin-Buch, Robert-Rössle-Str. 10, 13125 Berlin, Germany.

[b] N.-L. Efrém
Karlsruhe Institute of Technology, Institute of Organic Chemistry, Organic Chemistry I
Fritz-Haber-Weg 6, 76131 Karlsruhe, Germany.

[c] N. Csorba, P. Ábrányi-Balogh, L. Petri, V. Di Lorenzo, T. V. Szalai, L. Mihalovits, J. Simon, G. M. Keserű
HUN-REN Research Centre for Natural Sciences (HUN-REN RCNS)
Magyar tudósok körútja 2, H-1117 Budapest, Hungary.
National Laboratory for Drug Research and Development, Medicinal Chemistry, Magyar tudósok krt. 2, H-1117 Budapest, Hungary.

[d] N. Csorba, P. Ábrányi-Balogh, V. Di Lorenzo, G. M. Keserű
Department of Organic Chemistry and Technology, Faculty of Chemical Technology and Biotechnology, Budapest
University of Technology and Economics, Műegyetem rkp. 3, H-1111 Budapest, Hungary.

[e] M. Amoussa, Z. Guo
Freie Universität Berlin, Institute of Pharmacy
14195 Berlin, Germany.

[f] F. Bo, J. Li
Shanghai Institute of Materia Medica (SIMM), Chinese Academy of Sciences, 555 Zuchongzhi Road, Zhang Jiang Hi-Tech Park, Pudong, Shanghai, China.

[g] Y. Roske, O. Daumke
Max Delbrück Center for Molecular Medicine in the Helmholtz Association (MDC), Structural Biology, Berlin, Germany.

[h] M. Nazaré
Bielefeld University, Department of Chemistry, Organic and Bioorganic Chemistry, Universitätsstraße 25, 33615 Bielefeld, Germany.

*E-mail: nazare@fmp-berlin.de, keseru.gyorgy@ttk.hu

Contents

Single-point inhibition values of sulfonyl fluorides (SF) and fluorosulfates (OSF) S3
Chemistry S6
Materials and Methods S6
Synthesis of SF9-14 .... S7
Biochemical assay for fragment screening and IC50 determination S14
Non-biased SF fragments SF3 and SF5 .... S15
Tunnel allosteric site-biased fragments SF10-SF12 .... S16
Non-covalent controls 13-14 .... S17
Mass spectrometry experiments S18
Covalent labeling of SHP2 .... S18
Intact mass spectrometry experiments S18
Digestion experiments S18
LC-MS/MS measurements S19
Intact MS results S22
Digestion results S23
Intact MS-based competition experiment of SF12a vs. SHP099 .... S29
Determination of k_{inact}/K_I by intact MS S31
Stability assessment of the biased fragments S34
Structure analysis S35
Binding pose prediction S35
SHP2 overexpression and purification S35
Co-crystallization and structure determination S35
Binding poses of SF10-12 and comparison to drug-like tunnel-targeting allosteric SHP2 inhibitor S37
Analysis of SF-reactive amino acids in co-crystal structures of allosteric tunnel site-targeting SHP2 inhibitors S39
References S41

Single-point inhibition values of sulfonyl fluorides (SF) and fluorosulfates (OSF)

Table S1: Overview of SHP2[FL] inhibition data in single-point determination or IC_{50} values for all tested sulfonyl fluoride (SF) fragments, and where applicable, for their fluorosulfate (OSF) matched molecular pairs.

ID	SF Compound	% Inhibition @ 100 µM / IC_{50} [µM][a]	% Inhibition @ 100 µM[a] for OSF matched pair
**SF1**	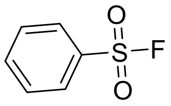	17±6%	39±6%
**SF2**	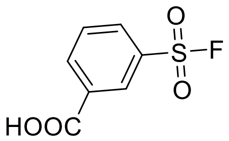	6±0%	Not available
**SF3**	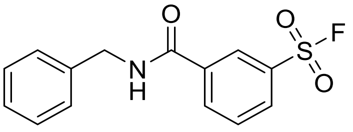	25±2%	32±6%
**SF4**	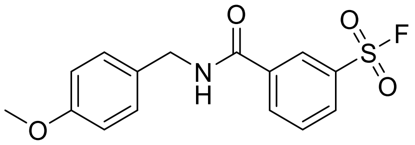	13±2%	Not available
**SF5**	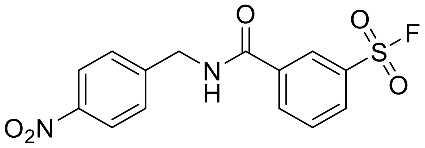	32±3%	8±5%
**SF6**	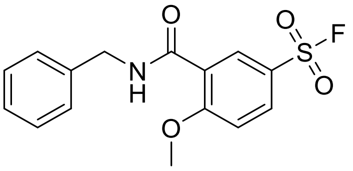	0%	Not available
**SF7**	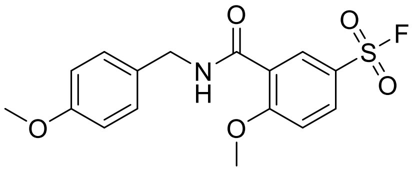	0%	Not available
**SF8**	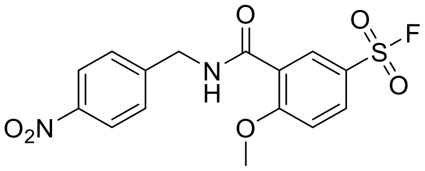	0%	Not available

**SF9**	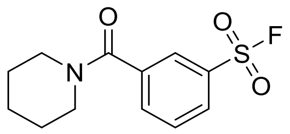	IC_{50} 8.2±1.9	Not available
**SF10**	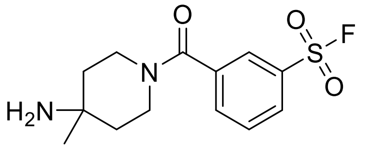	IC_{50} 190±40	Not available
**SF11**	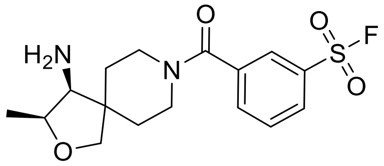	IC_{50} 160±20	Not available
**SF12a**	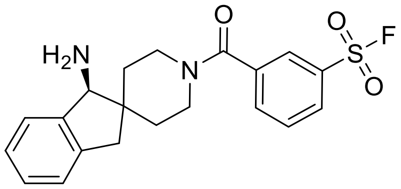	IC_{50} 5.8±1.0	11%±9%
**SF12b**	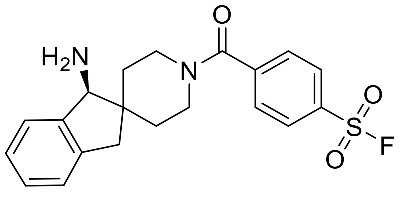	IC_{50} 13±5	7%±5%
**SF14**	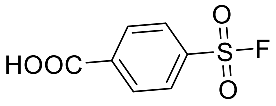	11±1%	Not available
**SF15**	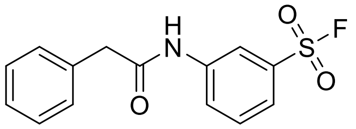	13±2%	Not available
**SF16**	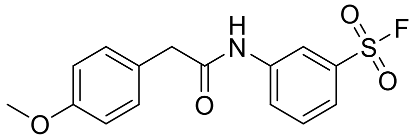	8±3%	Not available
**SF17**	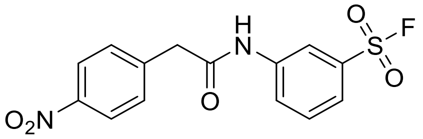	0%	Not available

**SF18**	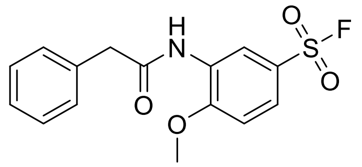	0%	Not available
**SF19**	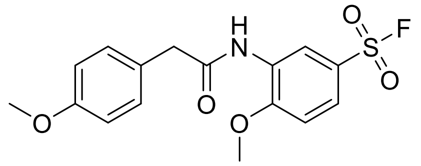	0%	Not available
**SF20**	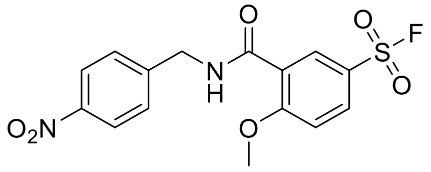	0%	Not available

[a] DiFMUP phosphatase assay[1] was used to determine % inhibition (at 100 μM inhibitor concentration) or inhibitor titration for IC_{50} determination against $SHP2^{FL}$ after 1 h pre-incubation at 37 °C. Values are mean ± S.D from technical triplicates. A detailed description of the experiment is provided below.

Chemistry

Materials and Methods All chemicals were purchased from commercial suppliers: Sigma-Aldrich, BLDPharm, Alfa Aesar, TCI Chemicals, ChemDiv, and Enamine. They were used as received unless otherwise specified. NMR spectra were recorded at either 295 K (300 MHz) or 300 K (600 MHz) at either Bruker AV 300 (300 MHz, 75 MHz) or Bruker AV 600 (600 MHz, 151 MHz) spectrometers. Chemical shifts are reported in ppm (δ) referenced to residual solvent peak such as DMSO (^1H NMR: 2.50 ppm) and $CHCl_3$ (^1H NMR: 7.26 ppm). LC/MS analysis was performed on an Agilent InfinityLab series LC/MS HPLC system with an Agilent Infinity II 1260 DAD coupled to an Agilent 6120 single quadrupole mass spectrometer (ESI-SQ) equipped with a Thermo Fisher Scientific Accucore C18 column, 2.1 x 30 mm, 2.6 µm. Method: ESI+, flux: 0.8 ml/min, 5-95% ACN in H_2O + 0.1% formic acid (FA), total runtime: 2.5 min or 8 min. High resolution mass spectra were recorded on an Agilent 6530 accurate-mass Q-ToF LC/MS linked to Agilent Technologies HPLC 1260 Infinity II. The HPLC was equipped with an Agilent Poroshell 120, C18 column, 2.1 x 100 mm, 1.8 µm. Method: ESI+, flux: 0.6 ml/min, 5-99% ACN in H_2O + 0.1% FA, total runtime: 4.5 min. Preparative HPLC was performed on a Gilson PLC 2250 with a Macherey-Nagel VP 250/21 Nucleodur 100-7 C18Ec column (30 mL/min flow). Purity and characterization of all final compounds was established by a combination of LC-MS, LC-HRMS and NMR analytical techniques. All tested compounds were found to be >95% pure by LC/MS and HRMS analysis.

Synthesis of SF9-14

The synthesis of the non-biased SF fragments has been reported elsewhere.[2]

3-(Piperidine-1-carbonyl)benzenesulfonyl fluoride (SF9)

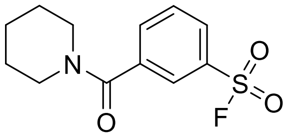

To a stirred solution of 3-(fluorosulfonyl)benzoic acid (30 mg, 147μmol, 1.00 eq.) in anhydrous ACN (3 mL), piperidine (44 μL, 441 μmol, 3.00 eq.) and HATU (67 mg, 176 μmol, 1.20 eq.) were added. The mixture was stirred at r.t. for 16 h. The mixture was filtered and purified by preparative HPLC (C18 column, solvent A: H_2O/0.1% FA, solvent B: ACN/0.1 FA. Gradient 15-75% solvent B over 20 min). 3-(Piperidine-1-carbonyl)benzenesulfonyl fluoride **SF9** was obtained as a colorless amorphous powder after lyophilization (16 mg, 58 μmol, 40 %).

LC-MS (ESI) (*m/z*): 272.1 $[M+H]^+$; HRMS (ESI) (*m/z*): calcd for $C_{12}H_{15}FNO_3S$ 272.0751 $[M+H]^+$; found 272.0746 $[M+H]^+$. ^{1}H NMR (300 MHz, CD_3CN) δ 8.10 (dt, *J* = 7.7, 1.7 Hz, 1H), 8.03 (t, *J* = 1.7 Hz, 1H), 7.90 – 7.80 (m, 2H), 7.76 (tt, *J* = 7.7, 0.8 Hz, 1H), 3.64 (s, 2H), 3.27 (s, 2H), 1.74 – 1.56 (m, 4H), 1.50 (s, 2H) ppm. ^{13}C NMR (75 MHz, CD_3CN) δ 167.67, 139.85, 135.13, 133.73, 133.41, 131.36, 129.80, 127.47, 49.27, 43.64, 26.88, 26.12 41, 25.01 ppm. ^{19}F NMR (282 MHz, CD_3CN) δ 142.06 ppm.

3-(4-Amino-4-methylpiperidine-1-carbonyl)benzenesulfonyl fluoride (SF10)

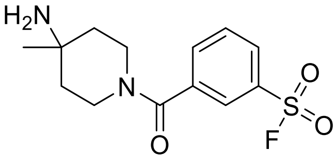

Step 1: To a stirred solution of 3-(fluorosulfonyl)benzoic acid (40 mg, 196 μmol, 1.00 eq.) in ACN (3 mL), DIPEA (70 μL, 392 μmol, 2.00 eq.) and HATU (89 mg, 235 μmol, 1.20 eq.) were added. The mixture was stirred at r.t. for 30 min. *Tert*-butyl (4-methylpiperidin-4-yl)carbamate (46 mg, 216 μmol, 1.00 eq.) was added and the reaction mixture was stirred at r.t. for 16 h. The mixture was filtered and purified by preparative HPLC (C18 column, solvent A: H_2O/0.1% FA, solvent B: ACN/0.1% FA. Gradient 15-65% solvent B over 30 min). *Tert*-butyl (1-(3-(fluorosulfonyl)benzoyl)-4-methylpiperidin-4-yl)carbamate was obtained as a colorless amorphous powder after lyophilization (56 mg, 137 μmol, 70 %).

LC-MS (ESI) (*m/z*): 423.1 $[M+Na]^+$/ 345.1 ES+ $[M-56]^+$; HRMS (ESI) (*m/z*): calcd for $C_{18}H_{25}FN_2O_5SNa$ $[M+Na]^+$ 423.1360; found 423.1343. ^{1}H NMR (300 MHz, CD_3CN) δ 8.13 – 8.07 (m, 1H), 8.04 (t, *J* = 1.7 Hz, 1H), 7.89 – 7.82 (m, 1H), 7.76 (tt, *J* = 7.8, 0.8 Hz, 1H), 5.23 (s, 1H), 4.16 – 4.02 (m, 1H), 3.37 – 3.16 (m, 4H), 2.15 – 1.90 (m, 1H), 1.65 – 1.39 (m, 1H), 1.39 (s, 9H), 1.29 (s, 3H) ppm. ^{13}C NMR (75 MHz, CD_3CN) δ 167.69, 155.72, 139.59, 135.18,

133.75, 133.43, 131.39, 129.89, 127.54, 118.26, 79.03, 51.44, 44.51, 38.88, 36.56, 36.05, 28.56, 26.45 ppm. ^{19}F NMR (282 MHz, CD_3CN) δ 142.40 ppm.

Step 2: *Tert*-butyl (1-(3-(fluorosulfonyl)benzoyl)-4-methylpiperidin-4-yl)carbamate (30 mg, 75 µmol, 1.00 eq.) was stirred in a 10:1 (v/v) mixture of anhydrous DCM/TFA (2.5 mL) for 1.5 h. The solvent was evaporated under a nitrogen flow. The residue was purified by preparative HPLC (C18 column, solvent A: H_2O/0.1% TFA, solvent B: ACN/0.1 TFA. Gradient 5-65% solvent B over 30 min). 3-(4-Amino-4-methylpiperidine-1-carbonyl)benzenesulfonyl fluoride 2,2,2-trifluoroacetate **SF10** was obtained as a colorless amorphous powder after lyophilization (22 mg, 52 µmol, 69 %). LC-MS (ESI) (*m/z*): 301.1 $[M+H]^+$/ 284.1 ES+ $[M-16]^+$; HRMS (ESI) (*m/z*): calcd for $C_{13}H_{17}FN_2O_3SNa$ 323.0836 $[M+Na]^+$; found 323.0825 $[M+Na]^+$. ^{1}H NMR (600 MHz, CD_3CN) δ 8.14 – 8.10 (m, 1H), 8.05 (t, *J* = 1.8 Hz, 1H), 7.89 – 7.85 (m, 1H), 7.79 (t, *J* = 7.8 Hz, 1H), 4.18 (s, 1H), 3.55 – 3.43 (m, 1H), 3.42 – 3.21 (m, 2H), 1.91 – 1.76 (m, 4H), 1.45 (s, 3H) ppm. ^{13}C NMR (151 MHz, CD_3CN) δ 168.18, 161.48, 161.27, 139.11, 135.30, 133.94, 133.78, 131.64, 130.28, 127.69, 118.34, 54.56, 44.22, 38.77, 35.90, 35.22, 22.74 ppm. ^{19}F NMR (564 MHz, CD_3CN) δ 64.54, -76.04 (TFA) ppm.

3-((3*S*,4*S*)-4-Amino-3-methyl-2-oxa-8-azaspiro[4.5]decane-8-carbonyl)benzenesulfonyl fluoride (SF11)

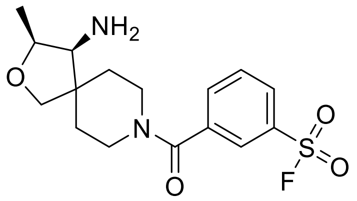

To a stirred solution of 3-(fluorosulfonyl)benzoic acid (75 mg, 367 µmol, 1.00 eq.) in anhydrous DMF (2 mL), DIPEA (65 µL, 367 µmol, 1.00 eq.) and HATU (140 mg, 367 µmol, 1.00 eq.) were added. The mixture was stirred at r.t. for 15 min and was then added dropwise to an ice-cooled solution of (3S,4S)-3-methyl-2-oxa-8-azaspiro[4.5]decan-4-amine dihydrochloride (89 mg, 367 µmol, 1.00 eq.) and DIPEA (195 µL, 1.10 mmol, 3.00 eq.) in anhydrous DMF (2 mL). The mixture was allowed to warm to r.t. over 1 h. Volatiles were removed by lyophilization. The crude material was purified by preparative HPLC (C18 column, solvent A: H_2O/0.1% FA, solvent B: ACN/0.1% FA. Gradient 5-65% solvent B over 40 min.) to afford 3-((3S,4S)-4-amino-3-methyl-2-oxa-8-azaspiro[4.5]decane-8-carbonyl)benzenesulfonyl fluoride **SF11** as a colorless amorphous powder after lyophilization (40 mg, 107 µmol, 29 %). LC-MS (ESI) (*m/z*): 357.2 $[M+H]^+$; HRMS (ESI) (*m/z*): calcd for $C_{16}H_{22}FN_2O_4S$ 357.1279 $[M+H]^+$; found 357.1296 $[M+H]^+$. ^{1}H NMR (600 MHz, MeOD) δ 8.18 (m, 1H), 8.12 (t, *J* = 1.8 Hz, 1H), 7.93 (d, *J* = 7.7 Hz, 1H), 7.85 (t, *J* = 7.8 Hz, 1H), 4.44 – 4.32 (m, 1H), 4.32 – 4.21 (m, 1H), 3.98 – 3.90 (m, 1H), 3.84 – 3.78 (m, 1H), 3.66 – 3.49 (m, 1H), 3.44 – 3.38 (m, 1H), 3.29 – 3.09 (m, 2H), 1.92 – 1.66 (m, 4H), 1.29 (d, *J* = 6.4 Hz, 3H) ppm. ^{13}C NMR (151 MHz, MeOD) δ 164.58, 138.98, 135.30,

131.86, 130.80, 130.63, 127.94, 76.51, 75.23, 62.78, 46.48, 40.30, 35.64, 35.24, 30.79, 30.38, 14.93 ppm. ^{19}F NMR (564 MHz, MeOD) δ 63.94 ppm.

(*S*)-3-(1-Amino-1,3-dihydrospiro[indene-2,4'-piperidine]-1'-carbonyl)benzenesulfonyl fluoride (SF12a)

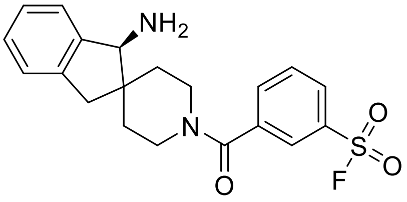

To a stirred solution of 3-(fluorosulfonyl)benzoic acid (40 mg, 196 µmol, 1.00 eq.) in anhydrous ACN (1 mL), DIPEA (34 µL, 196 µmol, 1.00 eq.) and HATU (89 mg, 235 µmol, 1.20 eq.) were added. The mixture was stirred at r.t. for 30 min and was then added dropwise to an ice-cooled solution of (*S*)-1,3-dihydrospiro[indene-2,4'-piperidin]-1-amine dihydrochloride (54 mg, 196 µmol, 1.00 eq.) and DIPEA (102 µL, 587 µmol, 3.00 eq.) in anhydrous ACN (2 mL) over 30 min. The mixture was allowed to warm to r.t. over 1 h, after which the solvent was removed by lyophilization. The crude material was purified by preparative HPLC (C18 column, solvent A: H_2O/0.1% FA, solvent B: ACN/0.1% FA. Gradient 15-65% solvent B over 40 min). (*S*)-3-(1-Amino-1,3-dihydrospiro[indene-2,4'-piperidine]-1'-carbonyl)benzenesulfonyl fluoride was obtained as a colorless amorphous powder after lyophilization (23 mg, 56 µmol, 12 %). LC-MS (ESI) (*m/z*): 372.1 $[M-16]^+$; HRMS (ESI) (*m/z*): calcd for $C_{20}H_{22}FN_2O_3S$ 389.1330 $[M+H]^+$; found 389.1345 $[M+H]^+$. ^{1}H NMR (600 MHz, CD_3CN) δ 8.13 (d, J = 7.9 Hz, 1H), 8.09 (t, J = 1.7 Hz, 1H), 7.90 (d, J = 7.7 Hz, 1H), 7.80 (t, J = 7.8 Hz, 1H), 7.51 (d, J = 7.5 Hz, 1H), 7.42 – 7.36 (m, 1H), 7.37 – 7.29 (m, 2H), 7.10 (broad s, 3H), 4.40 (m, 2H), 3.59 – 3.44 (m, 1H), 3.36 – 3.24 (m, 1H), 3.21 – 3.01 (m, 3H), 1.81 – 1.35 (m, 4H) ppm. ^{13}C NMR (151 MHz, CD_3CN) δ 168.08, 143.40, 139.35, 137.45, 135.38, 133.90, 133.74, 131.68, 131.12, 130.24, 128.31, 127.61, 126.85, 126.71, 65.06, 45.97, 45.57, 45.39, 39.95, 35.64, 34.81, 31.40, 30.60 ppm. ^{19}F NMR (564 MHz, CD3CN) δ 64.58 ppm

(*S*)-4-(1-Amino-1,3-dihydrospiro[indene-2,4'-piperidine]-1'-carbonyl)benzenesulfonyl fluoride (SF12b)

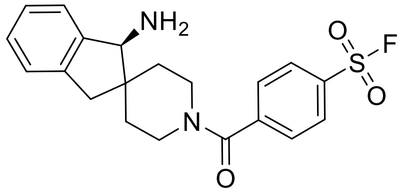

The compound was prepared according to procedure described for **SF12a**.

(*S*)-4-(1-Amino-1,3-dihydrospiro[indene-2,4'-piperidine]-1'-carbonyl)benzenesulfonyl fluoride **SF12b** was obtained as a colorless amorphous powder after lyophilization (40 mg, 98 µmol, 11 %). LC-MS (ESI) (*m/z*): 372.1 [M-16]$^+$; HRMS (ESI) (*m/z*): calcd for $C_{20}H_{22}FN_2O_3S$ 389.1330 [M+H]$^+$; found 389.1331 [M+H]$^+$. ^{1}H NMR (600 MHz, CD_3CN) δ 8.17 – 8.10 (m, 2H), 7.72 – 7.68 (m, 2H), 7.51 – 7.47 (m, 1H), 7.44 – 7.38 (m, 1H), 7.38 – 7.32 (m, 2H), 6.61 (broad s, 3H), 4.55 – 4.30 (m, 2H), 3.61 – 3.37 (m, 1H), 3.33 – 3.13 (m, 2H), 3.13 – 3.03 (m, 2H), 1.86 – 1.34 (m, 4H) ppm. ^{13}C NMR (151 MHz, CD_3CN) δ 168.36, 145.04, 144.95, 143.54, 137.13, 134.04, 133.87, 131.34, 129.99, 129.18, 128.41, 126.94, 126.82, 65.24, 45.81, 45.15, 39.87, 39.66, 35.55, 34.75, 31.51, 30.77 ppm. ^{19}F NMR (564 MHz, CD_3CN) δ 64.43 ppm.

(*S*)-(1-Amino-1,3-dihydrospiro[indene-2,4'-piperidin]-1'-yl)(3-(methylsulfonyl)phenyl)methanone (13a)

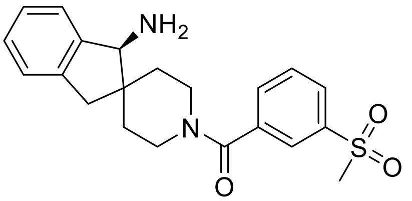

13a

The compound was prepared as described for **SF12a**. It was obtained as a colorless amorphous powder after lyophilization (30 mg, 77 µmol, 31 %).

LC-MS (ESI) (*m/z*): 385.2 [M+H]$^+$/368.2 [M-16]$^+$; HRMS (ESI) (*m/z*): calcd for $C_{21}H_{24}N_2O_3SNa$ 407.1400 [M+Na]$^+$; found 407.1392 [M+Na]$^+$. ^{1}H NMR (600 MHz, CD_3CN) δ 8.12 (s, 1H), 7.97 (dt, *J* = 7.7, 1.6 Hz, 1H), 7.93 (t, *J* = 1.8 Hz, 1H), 7.72 (dt, *J* = 7.7, 1.4 Hz, 1H), 7.68 (t, *J* = 7.7 Hz, 1H), 7.36 – 7.31 (m, 1H), 7.23 – 7.18 (m, 3H), 4.36 (s, 1H), 4.03 – 4.00 (m, 1H), 3.55 – 3.45 (m, 1H), 3.33 – 3.21 (m, 1H), 3.20 – 3.02 (m, 5H), 2.77 – 2.69 (m, 1H), 1.84 – 1.09 (m, 4H) ppm. ^{13}C NMR (151 MHz, CD_3CN) δ 168.77, 163.99, 142.32, 142.05, 139.07, 132.85, 130.78, 128.91, 128.59, 127.59, 126.46, 126.01, 125.19, 65.46, 47.59, 46.20, 45.58, 44.49, 40.54, 40.16, 39.92, 36.19, 35.41, 30.07, 29.19 ppm.

(*S*)-(1-Amino-1,3-dihydrospiro[indene-2,4'-piperidin]-1'-yl)(4-(methylsulfonyl)phenyl)methanone (13b)

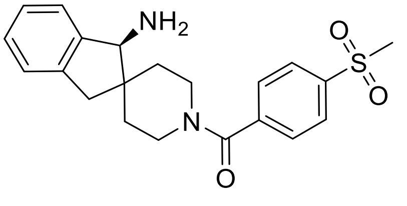

The compound was prepared as described for **13a**. It was obtained as a colorless amorphous powder after lyophilization (24 mg, 61 µmol, 25 %).

LC-MS (ESI) (*m/z*): 385.2 [M+H]$^+$/368.2 [M-16]$^+$; HRMS (ESI) (*m/z*): calcd for $C_{21}H_{24}N_2O_3SNa$ 407.1400 [M+Na]$^+$; found 407.1387 [M+Na]$^+$. ^{1}H NMR (600 MHz, CD_3CN) δ 7.97 (d, *J* = 8.0 Hz, 2H), 7.61 (d, *J* = 8.3 Hz, 2H), 7.34 – 7.29 (m, 1H), 7.20 (m, 3H), 4.36 (m, 1H), 3.97 (m, 1H), 3.46 (m, 1H), 3.34 – 3.13 (m, 1H), 3.08 (s, 3H), 2.73 (s, 1H), 2.74 – 2.62 (m, 2H), 2.35 (s, 12H), 1.87 – 1.54 (m, 2H), 1.45 – 1.08 (m, 2H) ppm. ^{13}C NMR (151 MHz, CD_3CN) δ 168.91, 143.11, 142.43, 141.92, 128.57, 128.54, 128.36, 127.54, 125.94, 125.03, 118.31, 65.63, 47.77, 46.06, 45.42, 44.51, 40.44, 40.17, 39.76, 38.87, 36.22, 35.47, 29.91, 29.07 ppm.

(*S*)-3-(1-Amino-1,3-dihydrospiro[indene-2,4'-piperidine]-1'-carbonyl)phenyl sulfurofluoridate (OSF12a)

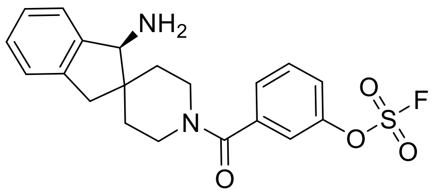

__Step 1:__ In an oven-dried flask, 3-hydroxybenzoic acid (50 mg, 362 µmol, 1.00 eq.) was dissolved in anhydrous ACN (2.5 mL). DIPEA (63 µL, 362 µmol, 1.00 eq.) and HATU (165 mg, 434 µmol, 1.20 eq.) were added. The mixture was stirred at r.t. for 5 min. (S)-1,3-Dihydrospiro[indene-2,4'-piperidin]-1-amine dihydrochloride (100 mg, 362 µmol, 1.00 eq.) was dissolved in anhydrous ACN (2.0 mL). DIPEA (126 µL, 724 µmol, 2.00 eq.) was added. The solution containing the activated acid was added. The mixture was stirred at r.t. for 16 h. The solvent was evaporated under reduced pressure. The crude material was purified by preparative HPLC (C18 column, solvent A: H_2O/0.1% FA, solvent B: ACN/0.1% FA. Gradient 5-55% solvent B over 30 min.). (*S*)-(1-Amino-1,3-dihydrospiro[indene-2,4'-piperidin]-1'-yl)(3-hydroxyphenyl)methanone **OSF12a** was obtained as a colorless amorphous powder after lyophilization (23 mg, 68 mmol, 19 %).

LC-MS (ESI) (*m/z*): 323.2 [M+H]$^+$/ 306.2 [M-16]$^+$; HRMS (ESI) (*m/z*): calcd for $C_{20}H_{23}N_2O_2$ 323.1754 [M+H]$^+$; found 323.1731 [M+H]$^+$. ^{1}H NMR (600 MHz, MeOD) δ 7.48 (d, *J* = 7.5 Hz, 1H), 7.42 – 7.35 (m, 2H), 7.33 (t, *J* = 7.3 Hz, 1H), 7.30 – 7.23 (m, 1H), 6.91 – 6.85 (m, 2H), 6.83 (s, 1H), 4.59 – 4.37 (m, 2H), 3.83 – 3.64 (m, 1H), 3.47 – 3.32 (m, 1H), 3.30 – 3.18 (m, 1H), 3.19 – 3.09 (m, 2H), 1.88 – 1.43 (m, 4H) ppm. ^{13}C NMR (151 MHz, MeOD) δ 172.48, 159.02, 143.49, 138.48, 138.08, 131.22, 130.95, 128.63, 127.05, 126.53, 118.56, 117.99, 114.66, 64.70, 46.10, 40.14, 36.38, 35.51, 32.17, 31.41 ppm.

Step 2: (*S*)-(1-Amino-1,3-dihydrospiro[indene-2,4'-piperidin]-1'-yl)(3-hydroxyphenyl)-methanone (15 mg, 47 µmol, 1.00 eq.) was dissolved in anhydrous ACN (1 mL). TEA (16 µL, 112 µmol, 2.40 eq.) was added. The mixture was stirred at r.t. for 10 min. The mixture was cooled to 0 °C and a solution of 1-(fluorosulfonyl)-2,3-dimethyl-1H-imidazol-3-ium trifluoromethanesulfonate (18 mg, 56 µmol, 1.20 eq.) in anhydrous ACN (0.5 mL) was added

dropwise to the amine over 30 min. The mixture was kept at 0°C for 2.5 h, then allowed to warm to r.t. and stirred for 14 h. The solution was filtered and purified by preparative HPLC (C18 column, solv A: H_2O/0.1% FA, solv B: ACN/0.1% FA), 10-65% B over 35 min. (*S*)-3-(1-amino-1,3-dihydrospiro[indene-2,4'-piperidine]-1'-carbonyl)phenyl sulfurofluoridate was obtained as a colorless amorphous powder after lyophilization (3 mg, 7 µmol, 16 %).

LC-MS (ESI) (*m/z*): 388.1 $[M-16]^+$; HRMS (ESI) (*m/z*): calcd for $C_{20}H_{22}FN_2O_4S$ 405.1279 $[M+H]^+$; found 405.1257 $[M+H]^+$. ^{1}H NMR (600 MHz, CD3CN) δ 7.61 (t, *J* = 7.9 Hz, 1H), 7.55 – 7.45 (m, 3H), 7.35 – 7.31 (m, 1H), 7.23 – 7.16 (m, 3H), 4.38 – 4.25 (broad s, 1H), 3.97 (s, 1H), 3.55 – 3.39 (m, 2H), 3.35 – 2.98 (m, 2H), 2.79 – 2.58 (m, 1H), 1.87 – 1.62 (m, 1H), 1.62 – 1.36 (m, 1H), 1.34 – 1.07 (m, 2H) ppm. ^{13}C NMR (151 MHz, CD3CN) δ 168.18, 150.87, 141.92, 140.56, 132.03, 128.34, 127.52, 125.92, 125.06, 122.74, 120.64, 65.63, 47.78, 46.20, 45.55, 40.64, 40.21, 39.91, 36.19, 35.47, 30.36, 29.86 ppm. ^{19}F NMR (564 MHz, CD_3CN) δ 37.04 ppm.

(*S*)-4-(1-Amino-1,3-dihydrospiro[indene-2,4'-piperidine]-1'-carbonyl)phenyl sulfurofluoridate (OSF12b)

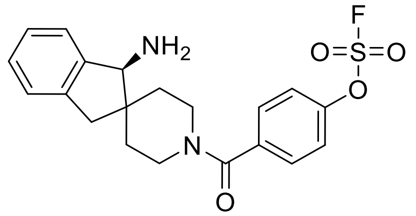

The compound was prepared according to the procedure described for **OSF12a**

Step 1: (*S*)-(1-Amino-1,3-dihydrospiro[indene-2,4'-piperidin]-1'-yl)(4-hydroxyphenyl)-methanone was obtained as a colorless amorphous powder after lyophilization (13 mg, 38 µmol, 11 %).

LC-MS (ESI) (*m/z*): 323.2 $[M+H]^+$/306.1 $[M-16]^+$; HRMS (ESI) (*m/z*): $C_{20}H_{23}N_2O_2$ 323.1754 $[M+H]^+$; found 323.1730 $[M+H]^+$. ^{1}H NMR (600 MHz, DMSO) δ 8.25 (s, 1H), 7.37 – 7.33 (m, 1H), 7.26 – 7.24 (m, 2H), 7.22 – 7.18 (m, 3H), 6.81 – 6.77 (m, 2H), 3.98 (s, 1H), 3.22 – 3.09 (m, 2H), 3.06 (d, *J* = 15.7 Hz, 1H), 2.69 (d, *J* = 15.6 Hz, 1H), 1.72 (td, *J* = 12.6, 4.4 Hz, 1H), 1.61 (td, *J* = 12.6, 4.4 Hz, 1H), 1.49 – 1.39 (m, 1H), 1.18 – 1.11 (m, 1H) ppm. 2 aliphatic protons are hidden under solvent peak. 13C NMR (151 MHz, DMSO) δ 169.06, 158.57, 144.06, 140.84, 131.44, 128.90, 127.43, 126.53, 126.35, 124.76, 124.31, 115.04, 114.83, 63.95, 46.26, 40.06, 39.06, 34.83, 28.68 ppm.

Step 2: (*S*)-4-(1-Amino-1,3-dihydrospiro[indene-2,4'-piperidine]-1'-carbonyl)phenyl sulfurofluoridate was obtained as a colorless amorphous powder after lyophilization (1.5 mg, 4 µmol, 13%).

LC-MS (ESI) (*m/z*): 388.1 $[M-16]^+$; HRMS (ESI) (*m/z*): $C_{20}H_{22}FN_2O_4S$ 405.1279 $[M+H]^+$; found 405.1270 $[M+H]^+$. ^{1}H NMR (600 MHz, CD_3CN) δ 7.58 (d, *J* = 8.4 Hz, 2H), 7.51 (d, *J* = 8.3 Hz,

2H), 7.40 – 7.37 (m, 1H), 7.24 – 7.19 (m, 3H), 4.36 (s, 1H), 4.07 (s, 1H), 3.50 (s, 1H), 3.32 – 3.04 (m, 2H), 2.83 – 2.70 (m, 1H), 1.80 – 1.45 (m, 4H) ppm. ^{13}C NMR (151 MHz, CD_3CN) δ 130.28, 128.89, 127.65, 126.08, 125.49, 122.24, 65.35, 40.18 ppm. ^{19}F NMR (564 MHz, CD_3CN) δ 36.99 ppm.

(*S*)-(1-Amino-1,3-dihydrospiro[indene-2,4'-piperidin]-1'-yl)(phenyl)methanone (14)

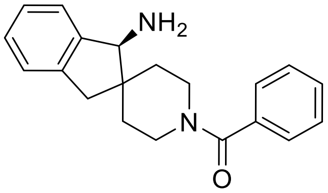

In an oven-dried flask, benzoic acid (35 mg, 287 µmol, 1.00 eq.) was dissolved in anhydrous DMF (1.5 mL). DIPEA (75 µL, 430 µmol, 1.50 eq.) and HATU (130 mg, 344 µmol, 1.20 eq.) were added. The mixture was stirred at r.t. for 30 min. (*S*)-1,3-Dihydrospiro[indene-2,4'-piperidin]-1-amine dihydrochloride (79 mg, 287 µmol, 1.00 eq.) was suspended in anhydrous DMF (2.5 mL). DIPEA (150 µL, 860 µmol, 3.00 eq.) was added. The mixture was cooled to 0°C. The solution containing the activated acid was added dropwise to this solution over 1h. The mixture was stirred at r.t. for 16 h. DMF was evaporated under reduced pressure. The residue was taken up in DCM (10 mL). The organic layer was washed with water (5 mL). The organic layer was dried over Na_2SO_4, filtered and concentrated under reduced pressure. The crude material was purified by preparative HPLC (C18 column, solvent A: H_2O/0.1% TFA, solvent B: ACN/0.1 TFA. Gradient 10-85% solvent B over 40 min.). The product was obtained as a colorless amorphous powder after lyophilization (43 mg, 138 µmol, 48 %).
LC-MS (ESI) (*m/z*): 307.2 $[M+H]^+$/209.2 $[M-16]^+$; HRMS (ESI) (*m/z*): calcd for $C_{20}H_{22}N_2ONa$ 329.1624 $[M+Na]^+$; found 329.1610 $[M+Na]^+$. ^1H NMR (600 MHz, MeOD) δ 7.54 – 7.45 (m, 6H), 7.45 – 7.38 (m, 2H), 7.36 (t, J = 7.4 Hz, 2H), 4.63 – 4.52 (m, 1H), 4.52 – 4.40 (m, 2H), 3.86 – 3.62 (m, 1H), 3.52 – 3.37 (m, 1H), 3.33 – 3.10 (m, 2H), 1.93 – 1.41 (m, 4H) ppm. ^{13}C NMR (151 MHz, MeOD) δ 172.51, 143.46, 138.52, 136.89, 131.19, 129.79, 128.62, 127.87, 127.02, 126.54, 64.68, 46.11, 46.00, 40.24, 40.15, 36.34, 35.54, 32.14, 31.37 ppm.

Biochemical assay for fragment screening and IC50 determination

The inhibitory activity of the non-biased SF fragment library[2] and of the target-biased SFs against SHP2^{FL} (amino acids 1525 with N-terminal GS from TEV protease cleavage site) was evaluated *in vitro* by measuring its catalytic activity using DiFMUP (Sigma, #SML2821) as the surrogate substrate in a modified fluorescence-based assay.[1, 3] The phosphatase reactions were performed in a 384-well, black polystyrene, flat bottom, non-binding surface plate (Greiner, Ref#781900) using a final reaction volume of 25 µL. SHP2^{FL} (5 nM) and *bis*-phosphorylated IRS-1 peptide (sequence: H_2N-LN(pY)IDLDLV(dPEG8) LST(pY)ASINFQK-amide, ordered from GenScript, 2 µM) was incubated for 5 min at ambient temperature. The pre-activated protein master mix (24.5 µL) was added to the tested compounds (0.5 µL, in DMSO) – final concentrations varying from 0.01 to 2000 µM or 100 µM for single-point screening of the in-house SF fragment library–in the following assay buffer conditions: 60 mM HEPES, 75 mM NaCl, 75 mM KCl, 1 mM EDTA, 0.05% Tween-20 (pH=7.2). After 60 min incubation at 37 °C, DiFMUP (Sigma, Cat#SML2821, 200 µM) was added to the reaction wells and incubated for 30 min at 25 °C. The fluorescence signals were measured (Ex:360/Em:450) using a microplate reader (Molecular Devices SpectraMax iD5 Multimode Microplate Reader, San Jose, CA, USA). The inhibitor dose-response curves were analyzed in GraphPad Prism 8.0.1 (GraphPad Software, La Jolla, CA, USA) using a normalized nonlinear regression model to determine IC_{50} values (with 95% confidence intervals). Data was normalized to control conditions, where the negative control (containing DMSO and buffer only) was defined as 100% inhibition and the positive control (containing DMSO and protein without inhibitor) was defined as 0% inhibition. Normalized values were then fitted to a sigmoidal dose-response (variable slope) curve to calculate the half-maximal inhibitory concentration (IC_{50}) for each compound. All measurements were performed in triplicates, and each experiment was independently repeated at least twice.

Non-biased SF fragments SF3 and SF5

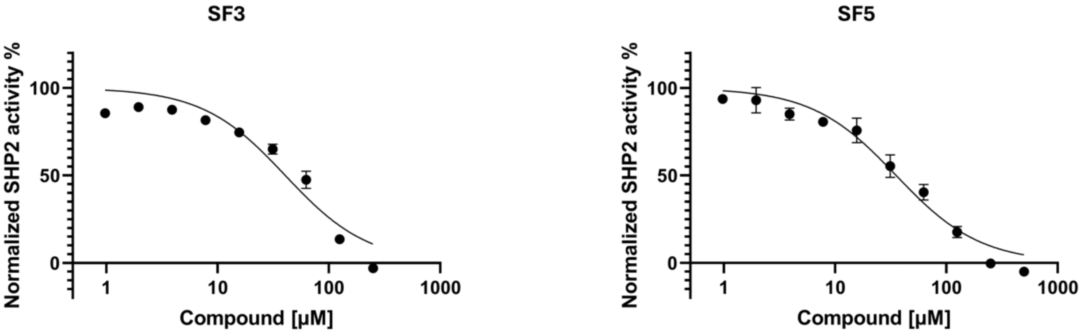

Figure S1: Titration curves of the non-biased sulfonyl fluoride (SF) fragments **SF3** and **SF5** against $SHP2^{FL}$ in presence of DiFMUP and the activating bis-phosphorylated IRS-1 peptide. Errors bars represent standard deviation (SD) from biological triplicates.

SF3: IC_{50} = 41 ± 11 µM (R^2=0.920)

SF5: IC_{50} = 35 ± 6 µM (R^2=0.962)

Tunnel allosteric site-biased fragments SF10-SF12

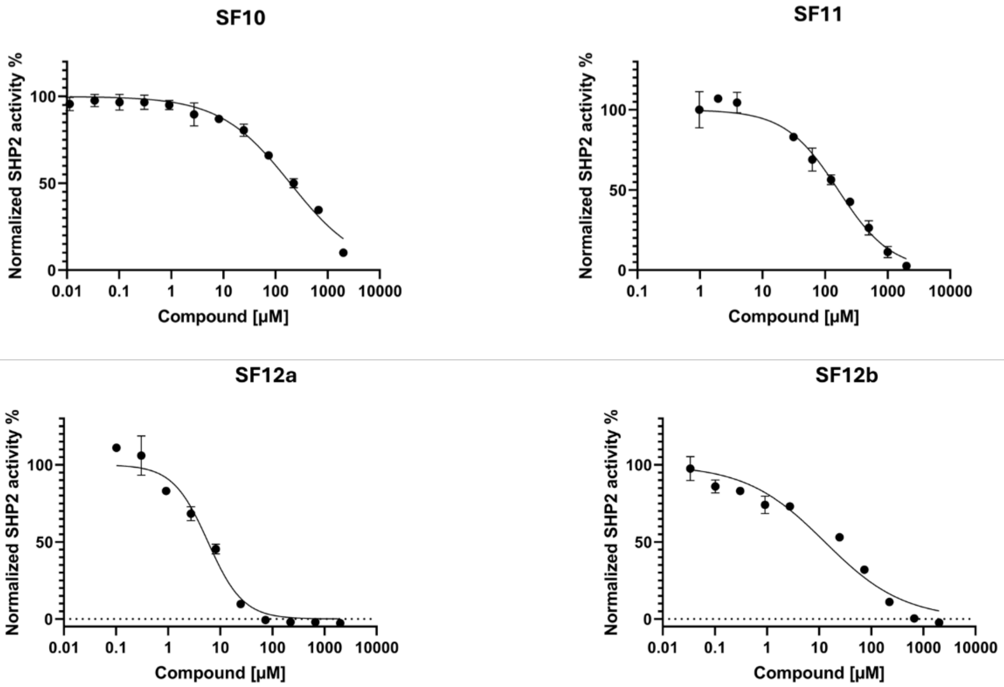

Figure S2: Titration curves of the tunnel allosteric site-biased sulfonyl fluoride (SF) fragments **SF10**, **SF11**, **SF12a** and **SF12b** against $SHP2^{FL}$ in presence of DiFMUP and the activating bis-phosphorylated IRS-1 peptide. Errors bars represent standard deviation (SD) from biological triplicates.

SF10: IC_{50} = 190 ± 40 µM (R^2=0.975)

SF11: IC_{50} = 160 ± 20 µM (R^2=0.977)

SF12a: IC_{50} = 5.8 ± 1.0 µM (R^2=0.977)

SF12b: IC_{50} = 13 ± 5 µM (R^2=0.955)

Non-covalent controls 13-14

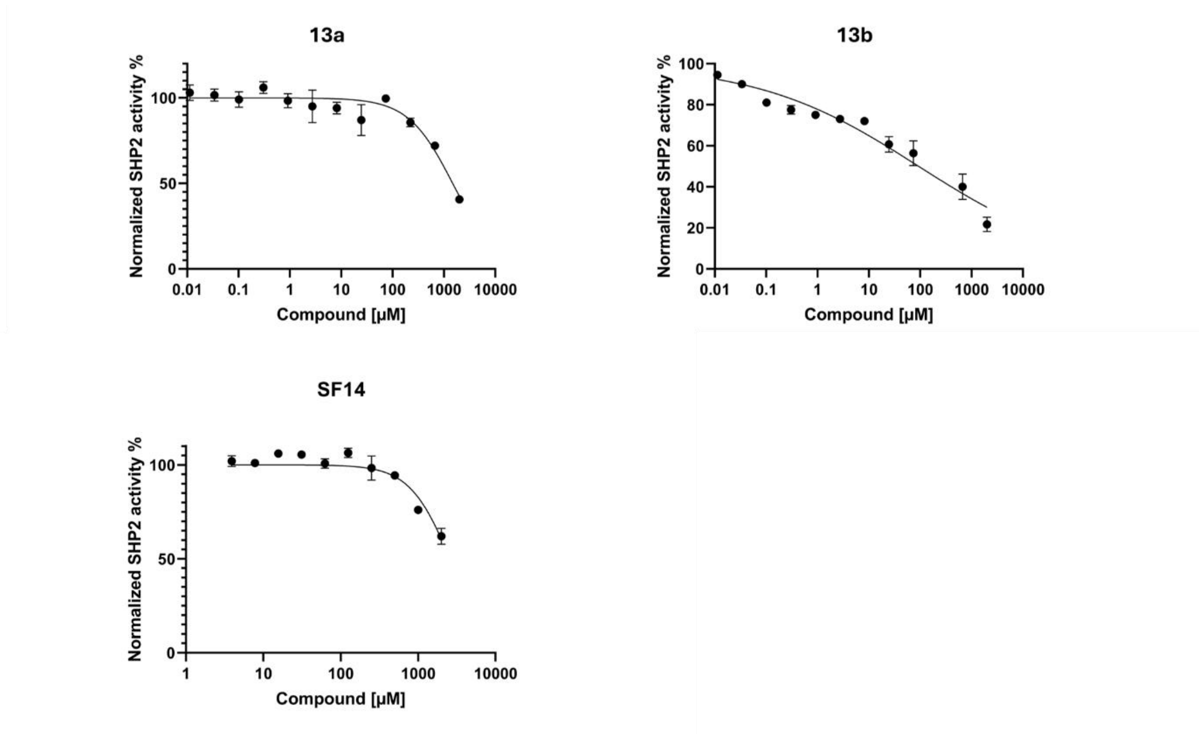

Figure S3: Titration curves of the non-covalent tunnel allosteric site-biased fragments **SF13a, SF13b** and **14** against SHP2^{FL} in presence of DiFMUP and the activating bis-phosphorylated IRS-1 peptide. Errors bars represent standard deviation (SD) from biological triplicates.

13a: IC_{50} > 1000 µM

13b: IC_{50} = 93± 36 µM (R^2=0.937)

14: IC_{50} > 1000 µM

Mass spectrometry experiments

Covalent labeling of SHP2

The protein was diluted with HEPES buffer (60 mM HEPES, 75 mM NaCl, 75 mM KCl, 1 mM EDTA (pH=7.2) to 1 mg/mL. To a microcentrifuge tube (Eppendorf Protein LoBind tube, PCR clean, withstand, capacity 0.5 mL) 1 µL of 42.5 mM fragment solution in DMSO was added in each tube and diluted with the protein solution to 50 µL (protein/compound ratio was 1:50 and the final sample contained 2 % DMSO). The samples were incubated at 37 °C for 60 minutes, then buffer exchange was performed three times with Sartorius Vivaspin 500 10000 MWCO at 15000 g for 7 minutes each time to remove the excess of the compounds. Afterwards, the concentrations were set to 1 mg/mL.

Intact mass spectrometry experiments

Next, 5 µL of the samples were transferred into microvials, diluted with MilliQ water to 0.2 mg/mL and the labeling was analyzed in intact mass spectrometry by using a Triple TOF 5600+ hybrid Quadrupole-TOF LC/MS/MS system (Sciex, Singapore, Woodlands) equipped with a DuoSpray IonSource coupled with a Shimadzu Prominence LC20 UFLC (Shimadzu, Japan) system consisting of binary pump, an autosampler and a thermostated column compartment, equipped with a Phenomenex SecurityGuard Widepore C4 4x3mm cartridge. The separation was achieved using mobile phase A (5% ACN in 0.1% FA) and B (95% ACN, 5% water 0.1% FA) using a gradient elution. A 4 min gradient (both in solvent composition and flow rate) was used with an initial flow of 0.5 mL/min and 10% eluent B. A 2 min linear increase was applied to reach the final flow of 1 mL/min and maximum eluent composition of B at 65%. These parameters were held for 0.5 min, and a 0.5 min linear gradient was used to reach the initial flow rate and eluent composition. This was followed by a 1 min equilibrating part. Data acquisition and processing were performed using Analyst TF software version 1.7.1 (AB Sciex Instruments, CA, USA).

Digestion experiments

After the labeling was confirmed by intact MS, 25 µL of the sample and 5 µL 0.2% (w/V) RapiGest SF (Waters, Milford, USA) solution buffered with 50 mM ammonium bicarbonate were mixed (pH=7.8) and 3 µL of 45 mM dithiothreitol (DTT) in 100 mM NH_4HCO_3 were added and kept at 37.5 °C for 30 min. After cooling the sample to room temperature, 3.5 µL of 100 mM iodoacetamide in 100 mM NH_4HCO_3 were added and placed in the dark at room temperature for 30 min. The reduced and alkylated protein was then digested by 3.5 µL (1 mg/mL) trypsin (the enzyme-to-protein ratio was 1:10) (Sigma, St Louis, MO, USA). The sample was incubated at 37 °C for overnight. To degrade the surfactant, 3 µL of formic acid (FA) (500 mM) solution was added to the digested protein sample to obtain the final 40 mM concentration (pH ≈ 2) and was incubated at 37 °C for 45 min. For LC-MS analysis, the acid

treated sample was centrifuged for 5 min at 13 000 rpm and the supernatant was pipetted into a microvial.

LC-MS/MS measurements

To get more precise information on the structure, samples were further analyzed by a Triple TOF 5600+ hybrid Quadrupole-TOF LC-MS/MS system (Sciex, MA, USA) equipped with a DuoSpray IonSource coupled with a Shimadzu Prominence LC20 UFLC (Shimadzu, Japan) system consisting of quaternary pump, an autosampler and a thermostated column compartment.

Data acquisition and processing were performed using Analyst TF software version 1.7.1 (AB Sciex Instruments, CA, USA). Chromatographic separation was achieved on the Discovery® BIO Wide Pore C-18-5 (250 mm × 2.1mm, 5 µm, 300 Å) HPLC column. Sample was eluted in gradient elution mode using solvent A (0.1% (V/V) FA in water) and solvent B (0.1% (V/V) FA in ACN). The initial condition was 5% B for 7 min, followed by a linear gradient to 90% B by 48 min, from 55 to 63 min 90% B was retained; and from 63 to 65 min back to initial condition with 5% eluent B and retained for 10 min. Flow rate was set to 0.2 ml/min. The column temperature was 40 °C and the injection volume was 15 µl. Nitrogen was used as the nebulizer gas (GS1), heater gas (GS2), and curtain gas with the optimum values set at 35, 35 and 35 (arbitrary units), respectively. The source temperature was 350 °C and the spray voltage was set to 5000 V.

Advanced Information Dependent Acquisition (IDA) mode was used on the TripleTOF 5600+ system to obtain MS/MS spectra on the 8 most abundant parent ions present in the TOF survey scan. In IDA LC-MS/MS experiments the mass spectra and tandem mass spectra were recorded in “high-sensitivity” mode with a resolution of ~35,000 full-width half-maximum.

In the first period (positive TOF MS mode) the data were acquired in the mass range of m/z=300 to 2500, with 0.1 s accumulation time. The declustering potential value was set to 60 V. The intensity threshold for precursor ion selection in TOF survey scan mode was 1000 cps. In MS2 experiments (Product Ion scan mode) the mass range was m/z=50 to 3000, with an accumulation time of 0.1 s.

Initial data was handled with PeakView software (version 2.2, Sciex) and Sciex OS (version 3.4.5.828). Acquisition files were converted for open-source applications by MSConvert (version 3.0.25071, ProteoWizard). Bottom-up proteomics search was executed by SearchGUI (version 4.3.15, CompOmics) with four algorithms (Comet, Tide, MetaMorpheus, and Sage). Results were analyzed by PeptideShaker (version 3.0.11, CompOmics). Novel self-made software, developed with the Python programming language (version 3.11.0), pyOpenMS (version 3.3.0), and with the help of Spyder Integrated Development Environment (version

5.5.0), was used for the final evaluation. Graphs were preparativeeared with Interactive Peptide Spectral Annotator.

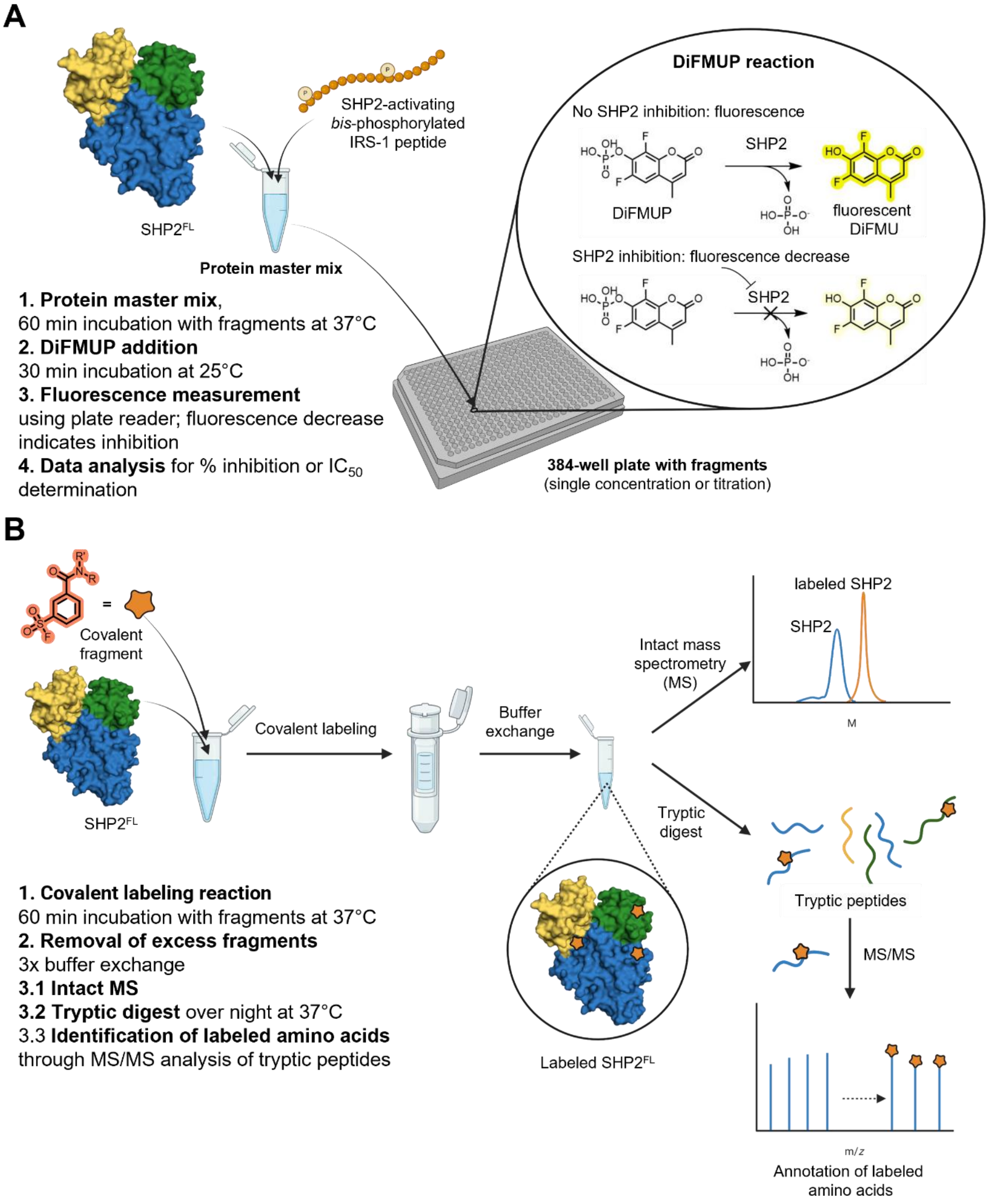

Figure S4: Schematic illustration of the workflow and assays used in the study to profile sulfonyl fluoride (SF) fragments. **A**: The inhibition of SHP2 phosphatase activity of all investigated compounds was determined using the DiFMUP phosphatase assay[1]. In brief, full-length (SHP2^{FL}) was activated by addition of an activating, bis-phosphotyrosyl IRS-1-derived peptide as described by Chen *et al.*.[3] The protein master mix containing SHP2 and the peptide was incubated with the assayed fragments in a 384 well plate, after which DiFMUP, a fluorogenic phosphatase substrate was added. The fluorescence intensity of the DiFMUP hydrolysis product (DiFMU) was measured using a plate reader after 30 min incubation at 25 °C. The fragments were used at 100 µM concentration for single-point determination (**Table S1**) or in a titration for IC_{50} determination of the most active compounds. To ensure comparability

of the IC_{50} values, identical incubation times were ensured for all tested compounds **B**: The most active SF fragments were incubated with SHP2 for 60 min at 37 °C, followed by buffer exchange using a centrifugal concentrator to remove excess fragments, and intact mass spectrometry (MS) analysis. To identify which amino acids were covalently modified by the SF fragments, labeled SHP2 was cleaved by incubation with trypsin protease over night at 37 °C. The tryptic peptides were subjected to MS/MS experiments that allow the annotation of labeled amino acids. Scheme was generated with Biorender.

Intact MS results

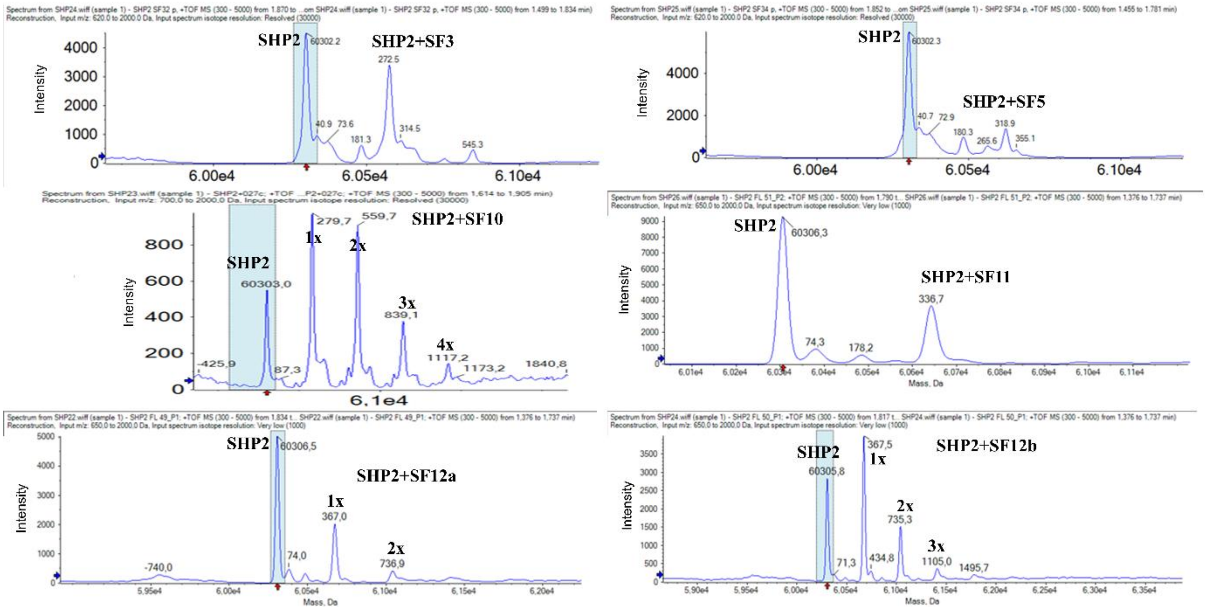

Figure S5: **Deconvoluted intact MS spectra of the covalent SF probes incubated with SHP2^{FL}.**

Table S2: Chemical formula and mass shift ∆MW of the sulfonyl fluoride (SF) probes.

Compound	∆MW [Da]	∆Form
**SF3**	273	$C_{14}H_{11}NO_3S$
**SF5**	318	$C_{14}H_{10}N_2O_5S$
**SF10**	280	$C_{13}H_{16}N_2O_3S$
**SF11**	336	$C_{16}H_{20}N_2O_4S$
**SF12a**	368	$C_{20}H_{20}N_2O_3S$
**SF12b**	368	$C_{20}H_{20}N_2O_3S$

Digestion results

SF3

MS/MS spectra of the peptides modified by **SF3** at IQNTGDYYDLYGGEK (A), FATLAELVQYYMEHHGQLK (B), FDSLTDLVEHYKK (C), KNPMVETLGTVLQLK (D), TWPDHGVPSDPGGVLDFLEEVHHK (E), and SGMVQTEAQYR (F). Labelled amino acids are ^{66}Y, ^{81}Y, ^{199}K, ^{426}H and ^{511}Y, respectively.

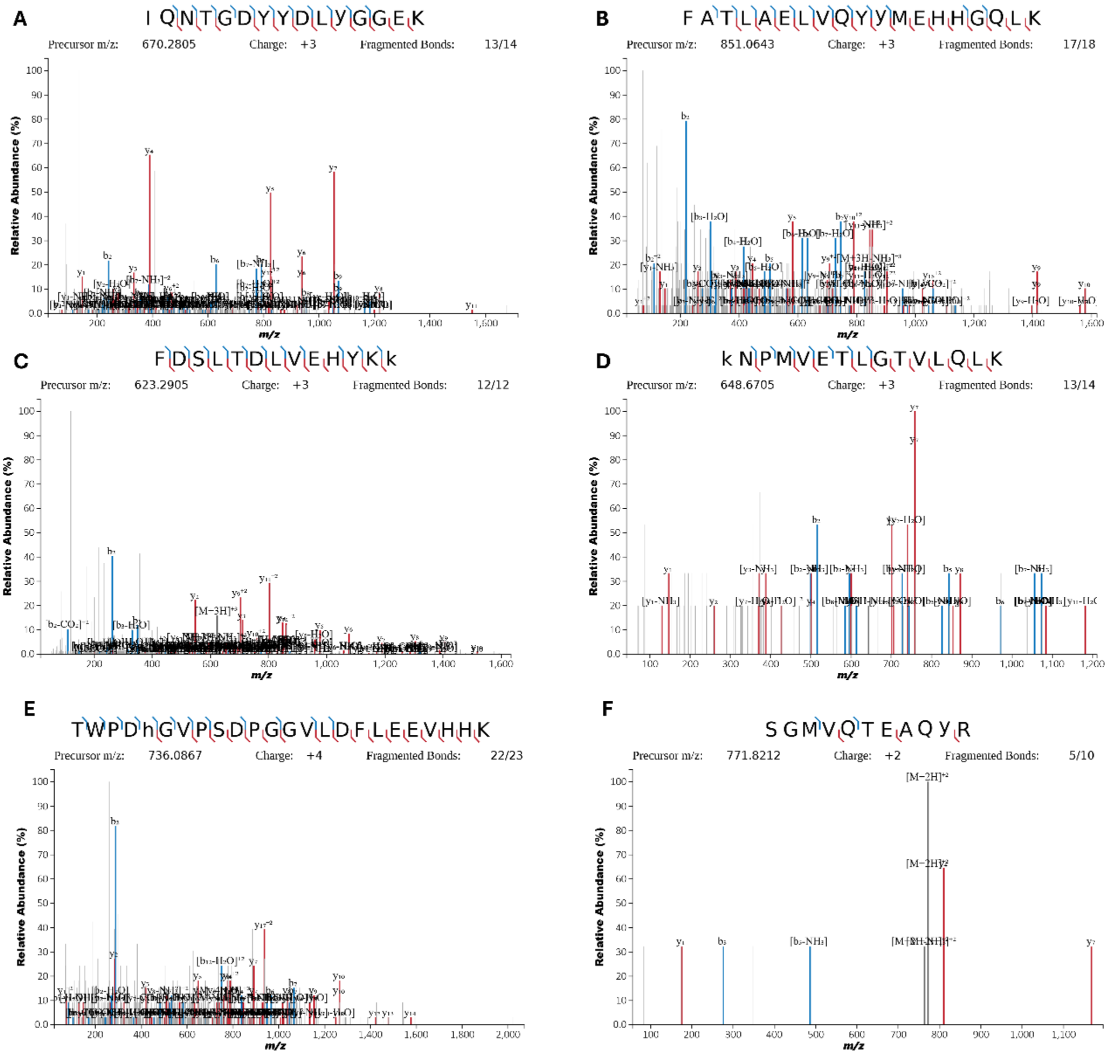

#	Sequence	Position start	Missed Cleavages	RT (min)	*m/z*
A	IQNTGDYYDLYGGEK	56	0	31.29	670.31
B	FATLAELVQYYMEHHGQLK	71	0	34.08	851.10
C	FDSLTDLVEHYKK	187	1	32.3	623.31
D	KNPMVETLGTVLQLK	199	1	34.63	648.70
E	TWPDHGVPSDPGGVLDFLEEVHHK	422	0	33.29	736.11
F	SGMVQTEAQYR	502	0	29.20	771.85

Figure S6: LC-MS/MS spectra of digested SHP2^{FL} after labeling by **SF3** and analytical data of the modified peptides detected in the peptide mapping analysis using tryptic digestion.

SF5

MS/MS spectra of the peptides IQNTGDYYDLYGGEK (A), FATLAELVQYYMEHHGQLK (B), FDSLTDLVEHYKK (C) and TWPDHGVPSDPGGVLDFLEEVHHK (D) modified by **SF5** at ^{66}Y, ^{81}Y, ^{198}K and ^{426}H, respectively.

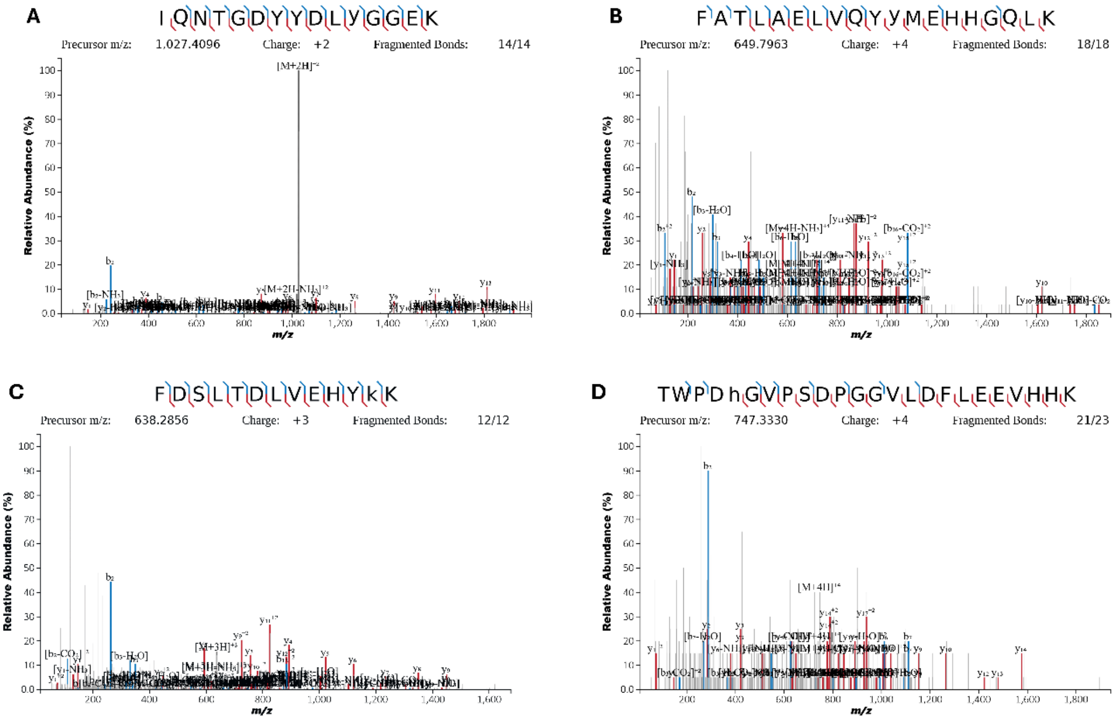

#	Sequence	Position start	Missed Cleavages	RT (min)	*m/z*
A	IQNTGDYYDLYGGEK	56	0	31.09	1027.41
B	FATLAELVQYYMEHHGQLK	71	0	34.90	649.82
C	FDSLTDLVEHYKK	187	1	32.13	638.31
D	TWPDHGVPSDPGGVLDFLEEVHHK	422	0	32.95	747.36

Figure S7: LC-MS/MS spectra of digested SHP2^{FL} after labeling by **SF5** and analytical data of the modified peptides detected in the peptide mapping analysis using tryptic digestion.

SF10

MS/MS spectra of the peptides IQNTGDYYDLYGGEK (A), YPLNC[CAM]ADPTSER (B), YDVGGGER (C) modified by **SF10** at ^{66}Y, ^{100}Y, ^{179}Y, respectively.

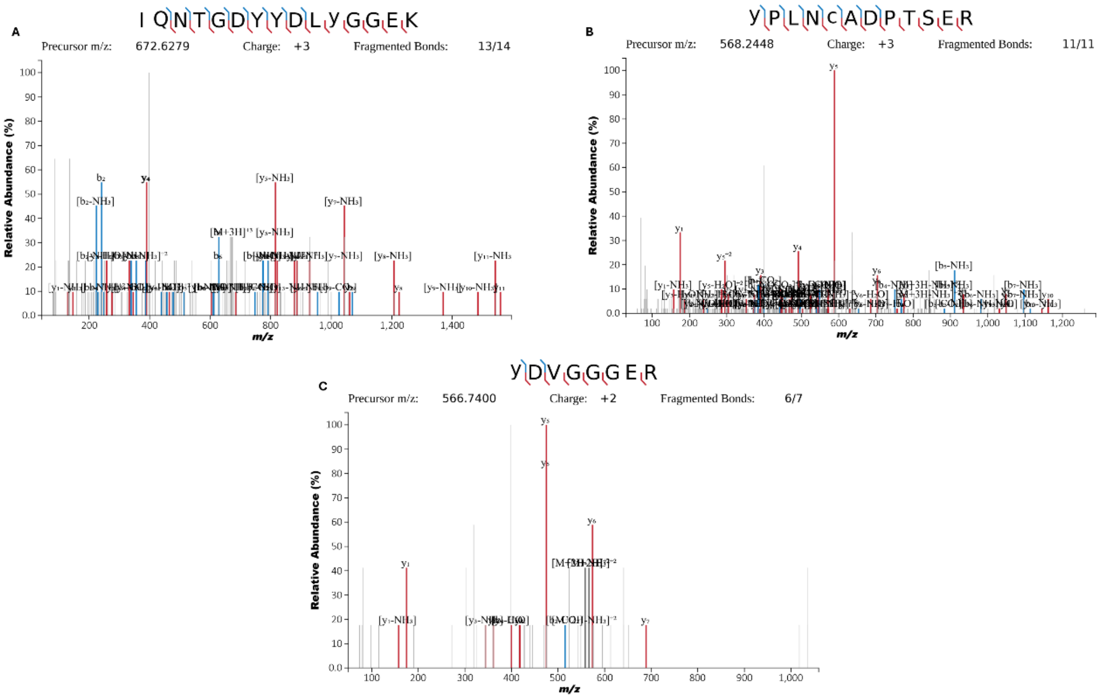

#	Sequence	Position start	Missed Cleavages	RT (min)	*m/z*
**A**	IQNTGDYYDLYGGEK	56	0	24.80	672.60
**B**	YPLNCADPTSER	100	0	22.04	568.20
**C**	YDVGGGER	179	0	20.35	566.70

Figure S8: LC-MS/MS spectra of digested SHP2^{FL} after labeling by **SF10** and analytical data of the modified peptides detected in the peptide mapping analysis using tryptic digestion.

SF11

MS/MS spectra of the peptides IQNTGDYYDLYGGEK (A), YDVGGGER (B), VIVMTTKEVER (C) and ELSKLAETTDK (D) modified by **SF11** at ^{66}Y, ^{179}Y, ^{235}K and ^{358}K, respectively.

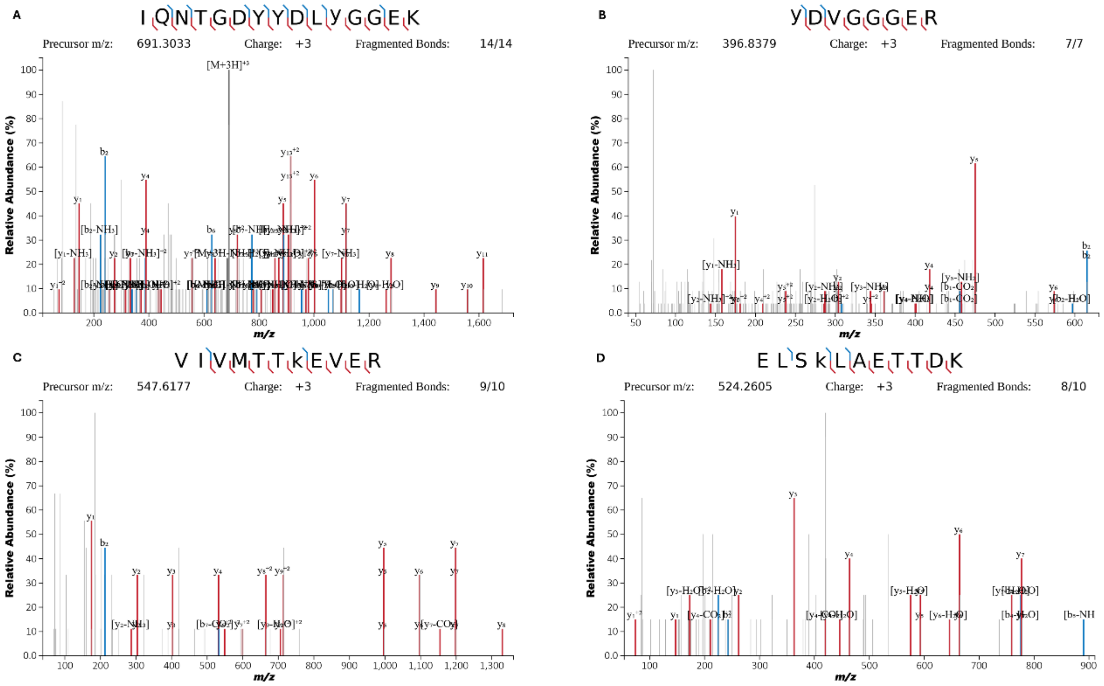

#	Sequence	Position start	Missed Cleavages	RT (min)	*m/z*
A	IQNTGDYYDLYGGEK	56	0	26.27	691.30
B	YDVGGGER	179	0	20.84	396.80
C	VIVMTTKEVER	352	1	23.07	547.60
D	ELSKLAETTDK	232	1	22.19	524.27

Figure S9: LC-MS/MS spectra of digested SHP2^{FL} after labeling by **SF11** and analytical data of the modified peptides detected in the peptide mapping analysis using tryptic digestion.

SF12a

MS/MS spectra of the peptides GVDC[CAM]DIDVPKTIQMVR and YPLNC[CAM]ADPTSER modified by **SF12a** at ^{492}K and ^{100}Y, respectively.

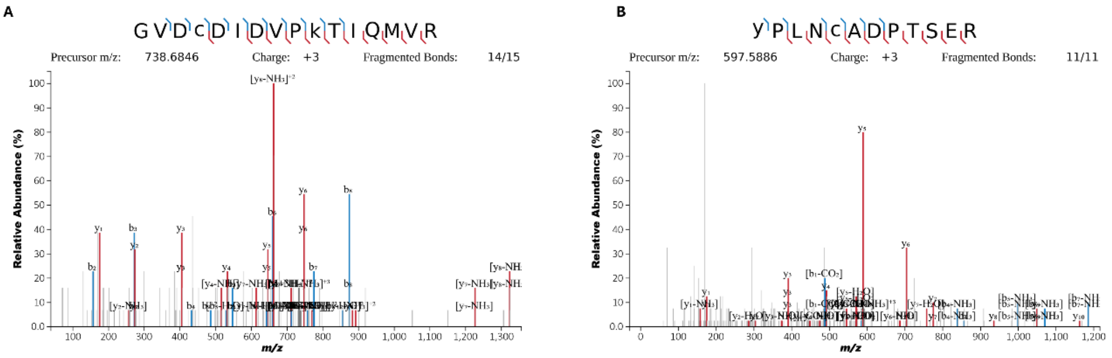

#	Sequence	Position start	Missed Cleavages	RT (min)	*m/z*
A	GVDCDIDVPKTIQMVR	483	1	28.25	738.70
B	YPLNCADPTSER	100	0	25.22	597.60

Figure S10: LC-MS/MS spectra of digested SHP2^{FL} after labeling by **SF12a** and analytical data of the modified peptides detected in the peptide mapping analysis using tryptic digestion.

SF12b

MS/MS spectra of the peptides GVDC[CAM]DIDVPKTIQMVR and IQNTGDYYDLYGGEK modified by **SF12b** at 492K and 66Y, respectively.

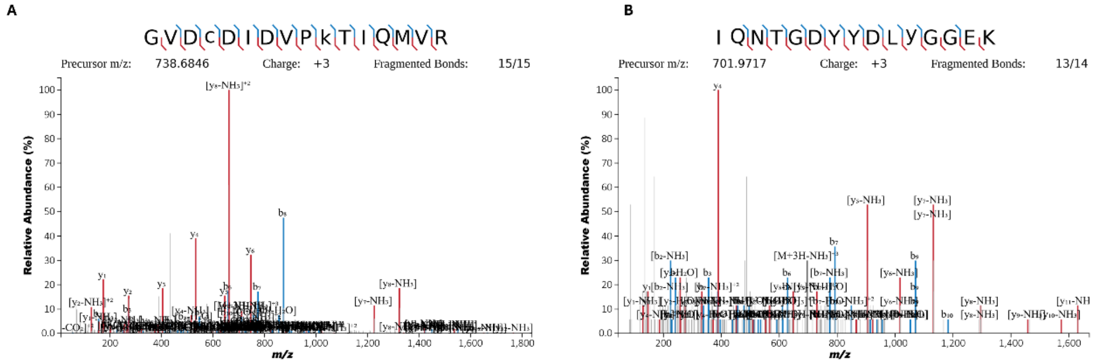

#	Sequence	Position start	Missed Cleavages	RT (min)	*m/z*
A	GVDCDIDVPKTIQMVR	483	1	28.37	738.70
B	IQNTGDYYDLYGGEK	56	0	28.37	702.00

Figure S11: LC-MS/MS spectra of digested SHP2^{FL} after labeling by **SF12b** and analytical data of the modified peptides detected in the peptide mapping analysis using tryptic digestion.

Intact MS-based competition experiment of SF12a vs. SHP099

Full-length SHP2 was diluted to a final concentration of 250 µg/mL in HEPES buffer (60 mM HEPES, 75 mM NaCl, 75 mM KCl, 1 mM EDTA; pH 7.2). A total of 0.5 µL of DMSO solution was added to the protein solution to obtain a final reaction volume of 25 µL as follows: For the covalent probe sample, 0.25 µL of fragment DMSO stock and 0.25 µL of pure DMSO were added. For the competitive sample, 0.25 µL of **SF12a** DMSO stock solution and 0.25 µL of **SHP099** DMSO stock solution were added. The final concentration of **SF12a** and **SHP099** in the reaction mixture was 100 µM. The samples were incubated at 37 °C for 1 h. Subsequently, the reactions were quenched by adding 2.5 µL of a 10% (V/V) formic acid solution. The incubations were performed in a 384-well black polystyrene flat-bottom non-binding surface plate (Greiner, Ref# 781900). The samples were transferred into microvials immediately before measurement and analyzed using the above-mentioned intact MS method. All experiments were performed in duplicate. The labeling ratio was determined from the ratio of the MS peak heights using PeakView software (version 2.2, Sciex).

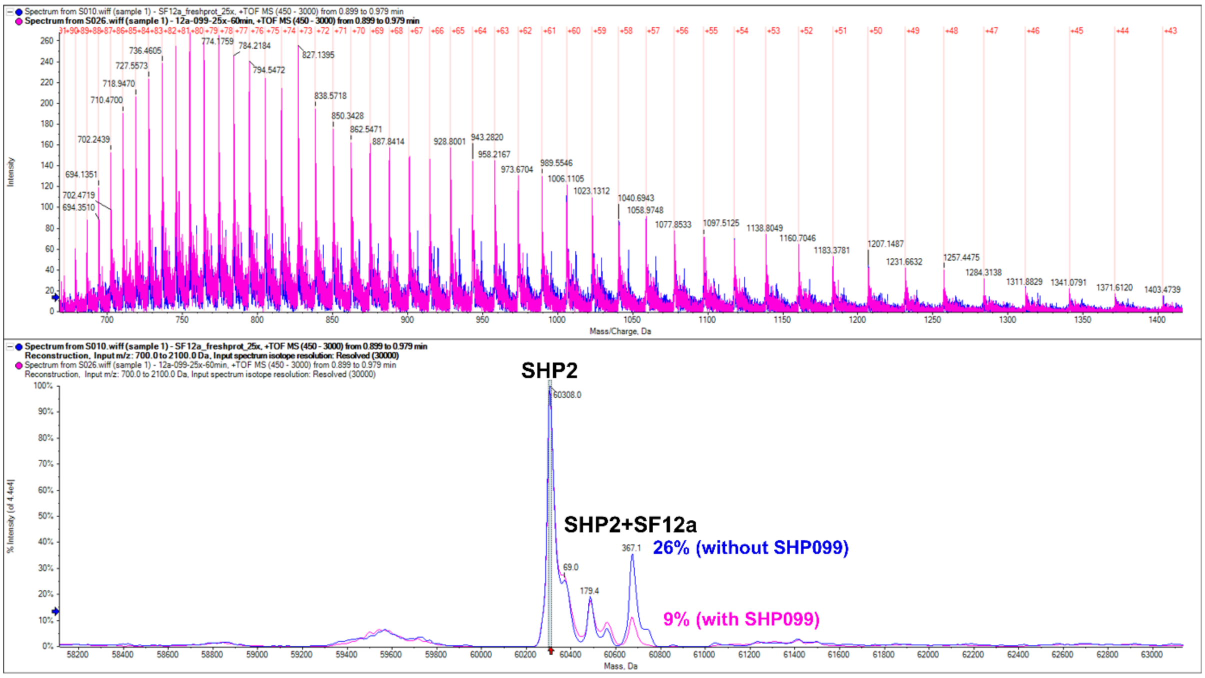

Figure S12: Difference in the intact MS labeling ratio (%) in presence (pink) and absence (blue) of the potent non-covalent tunnel-targeting allosteric SHP2 inhibitor **SHP099**[4] for labeling full-length SHP2 with **SF12a**, after incubation for 1 h at 37 °C.

Determination of k_{inact}/K_I by intact MS

Full-length SHP2 was diluted to a final concentration of 250 µg/mL in HEPES buffer (60 mM HEPES, 75 mM NaCl, 75 mM KCl, 1 mM EDTA; pH 7.2). To this solution, 0.3 µL of DMSO stock solutions of the fragments were added to give a final reaction volume of 15 µL. The incubations were performed at 25 °C, in a 384-well, black polystyrene, flat bottom, non-binding surface plate (Greiner, Ref#781900), transferred into microvials right before the measurement, and measured using the above-described intact MS method. For compound **SF12a** and **SF12b**, the final concentrations were 76, 220, and 660 µM, respectively. The incubation times for compound **SF12a** were 0, 60, 120, 360, 600, and 960 min, while those for compound **SF12b** were 0, 30, 60, 120 and 240 min. Subsequently, the reactions were quenched by adding 1.5 µL of 10% (V/V) formic acid. Each experiment was performed in duplicates. The ratio of the labeling was determined from the ratio of the MS peak heights handled with PeakView software (version 2.2, Sciex). After the identification of single occupancy levels in each experimental setup, those were plotted against time using GraphPad Prism Software (v8.0.1, GraphPad, La Jolla, San Diego, CA, USA). Based on experimental decay we k_{obs} (min^{-1}) was calculated. These values were then plotted against the concentration of the probe and K_I and k_{inact} were calculated directly from nonlinear regression according to the k_{obs} – c function shown in equation 1 (Eq. **1**).

$$k_{obs} = \frac{c \cdot k_{inact}}{c + K_I} \qquad \text{(Eq. 1)}$$

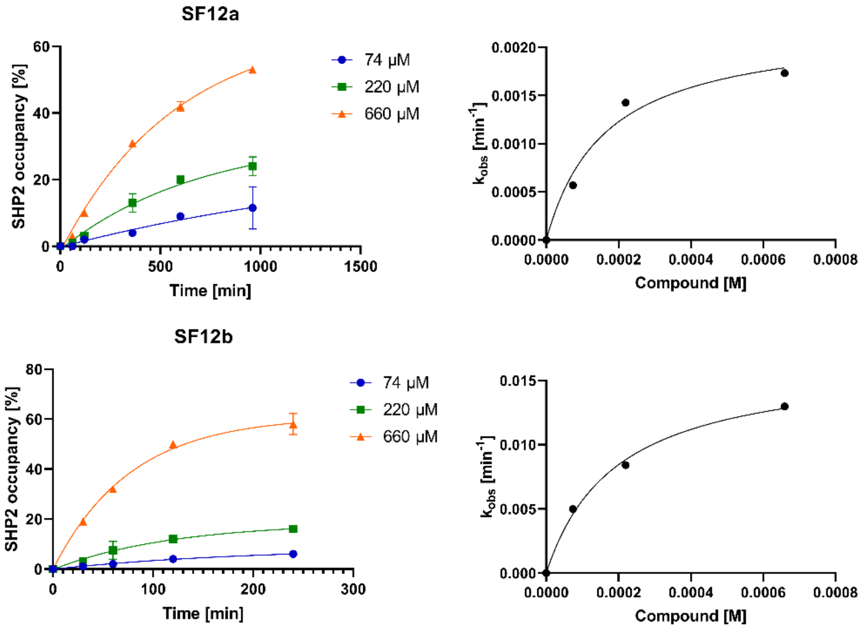

Compund [M]	k_{obs} [min^{-1}]
0	0
0.000074	0.000570
0.000220	0.001426
0.000660	0.001732

Compund [M]	k_{obs} [min^{-1}]
0	0
0.000074	0.004989
0.000220	0.008418
0.000660	0.012980

Figure S13: Determination of the k_{inact} and K_I parameters for the interaction of **SF12a** and **SF12b** with SHP2. The left panel shows time-dependent target occupancy at varying inhibitor concentrations (74, 220, 660 µM) that results in the observed first-order rate constants (k_{obs}), where the percent of covalently labeled SHP2 was assessed by MS and exponential one-phase decay regression was used (with fixed zero percent labelling at t = 0 min) to calculate the k_{obs} values. Next, calculated k_{obs} values were plotted against the compound concentration (right). The K_I and k_{inact} values were calculated directly from non-linear regression according to the k_{obs} – c function (**Eq. 1**), resulting the following values: **SF12a** k_{inact}=0.002 min^{-1}, K_I=164 µM; k_{inact}/K_I=12 M^{-1} min^{-1}; **SF12b** k_{inact} =0.017 min^{-1}, K_I=193 µM; k_{inact}/K_I=88 M^{-1} min^{-1}. The tables show the calculated k_{obs} values according to the covalent engagement of SHP2 at different measured concentrations of the covalent probes. Data shown are results of duplicate experiments.

Stability assessment of the biased fragments

HPLC-MS measurements were performed using a Shimadzu LCMS-2020 device equipped a positive–negative double ion source (DUIS±) and a quadrupole MS analyzer in the range of m/z 50–1000. The sample was eluted with gradient elution using eluent A (0.1% FA in H_2O) and eluent B (0.1% FA in ACN). The column temperature was always kept at 30 °C; the injection volume was 5 µL, and the flow rate was set to 1.5 mL/min. A Reprospher C18 (5 µm, 100 mm × 3 mm) column was used along with the following gradient. The initial condition was 0% B eluent, followed by a linear gradient to 100% B eluent by 13 min; from 13 to 14 min, 100% B eluent was retained. From 14 to 15 min, the initial condition with 0% B eluent was restored.

In a glass LC-MS vial, the internal standard (indoprofen, 1 µL, 10 mM in DMSO) and the buffer solution (98 µl; 60 mM HEPES, 75 mM NaCl, 75 mM KCl, 1 mM EDTA, pH=7.2) were mixed. To this solution the respective biased fragments were added (1 µL, 100 mM in DMSO) right before the measurement. The vial was mixed, then analyzed by HPLC-MS (5 µL injection volume) at intervals of 0, 1, 2, 3 h, and 20 h, respectively. The AUC (area under the curve) values were determined via integration of MS chromatograms and then corrected with the internal standard. The fragments' AUC values were subjected to ordinary least-squares (OLS) linear regression, and to compute the parameters (kinetic rate constant (k) and half-life time ($t_{1/2}$)), an Excel sheet was applied. The pseudo first-order kinetic constant ($k_{stability}$) was determined as means of duplicate determination (k_A and k_B). The half-life was determined using the equation $t_{1/2} = \ln 2/k_{stability}$.

Table S3: Determination of pseudo first-order kinetic constants ($k_{stability}$) and half-life ($t_{1/2}$) of the target-biased sulfonyl fluorides at pH 7.2 by measuring hydrolytic stability.

Compound ID	k_A [h^{-1}]	k_B [h^{-1}]	$k_{stability}$ [h^{-1}]	$t_{1/2}$ [h]
**SF10**	1.06	0.74	0.90	0.8
**SF11**	1.04	0.90	0.97	0.7
**SF12a**	0.24	0.10	0.17	4.1
**SF12b**	0.38	0.21	0.29	2.4

Structure analysis

Binding pose prediction

Protein structures with PDB IDs 6MDC[5] and 8T6D[6] were prepared with Maestro's Protein Preparation Workflow[7], at a pH of 7.4 using default settings, while ligands were prepared using LigPrep[7] at a pH range of 7.4±1.0. Receptor grids were set by using the ligand in the appropriate protein structure as reference, which included the tunnel site. Non-covalent docking was performed using Glide SP[8-9] using default settings, without constraints. Covalent docking was performed using CovDock[10] (Pose Prediction docking mode), targeting residue K492.

SHP2 overexpression and purification

Human SHP2 (aa 2-525), cloned into pQlinkH vector, was recombinantly overexpressed in *E.coli* Rosetta DE3 with a TEV-cleavable N-terminal His7-tag. Cells were grown in TB-medium at 37°C to an OD600 nm of 0.8 before lowering to temperature to 18°C for inducing expression by 500 µM isopropyl β-D-1-thiogalactopyranoside (IPTG) for overnight cultivation. Cells were harvested by 20 min centrifugation at 7000 rpm and were lysed by two freeze and thaw cycles in lysis buffer (1x PBS, 0.5 M NaCl, 5% Glycerol, 1 µg/mL Lysozyme, 0.1% Triton X-100, 2 µg/mL DNAseI, 250 µM Pefabloc) and final centrifugation of the lysate for 45 min at 10°C and 22500 rpm. The supernatant was supplemented with 15 mM imidazole and applied to an IMAC HP HisTrap column (Cytiva ®) equilibrated with buffer A (20 mM Tris pH 8, 0.5 M NaCl). His-tag bound SHP2 was eluted by an imidazole gradient with a maximum of 250 mM. Fractions with target protein were pooled, His-TEV-protease was added and dialyzed overnight at 4°C against buffer A. The TEV-cleaved SHP2 was re-applied to the HP HisTrap column and the flow through SHP2 fractions were collected and concentrated for application to size exclusion column HiLoad 16/600 Superdex S200 (Cytiva ®) in 20 mM Tris pH 8, 150 mM NaCl, 2 mM DTT running buffer. SHP2 fractions were pooled and concentrated to 12.7 mg/mL (211 µM). Purification success was checked by SDS PAGE.

Co-crystallization and structure determination

Purified SHP2$^{2\text{-}525}$ was combined with a final concentration of 0.7 mM compound **SF12a** containing 2% DMSO in total and crystalized using the sitting-drop vapor-diffusion method by mixing 200 nL of complex solution and equal volume of crystallization solution containing 20% PEG 3350, 0.2 NaF, 0.1 M Bis-Tris propane pH 8.5. Experiments were performed with a Gryphon pipetting robot (Matrix Technologies Co.) and Rock imager 1000 storage system (Formulatrix). Crystals appeared within 7 days and were transferred into a cryo-protection solution containing the crystallization solution supplemented with 20% ethylene glycol before flash freezing in liquid nitrogen. Diffraction data were recorded to 2.35 Å at BL14.1 at BESSY II (Helmholtz-Zentrum Berlin, HZB), processed and scaled using XDSapp.[11] The complex

structure was solved by molecular replacement with Phaser using the SHP2 structure pdb 5EHR as search model.[12] The structure and compound was manually adjusted using COOT[13] and iteratively refined using Refmac version 5.8.0267[14] to an Rwork of 24.3% and Rfree of 28.3% (**Table S4**).

Table S4: Data collection and refinement statistics.

	$SHP2_{(2\text{-}525)}$ + **SF12a** (hydrol.)
**Data collection**	
Space group	$P2_1$
Cell dimensions	
a, *b*, *c* (Å)	44.9, 212.5, 55.5
α, β, γ	90.0, 95.2, 90.0
Resolution (Å)*	48.0 – 2.35 (2.49-2.35)
R_{meas}*	23.8 (246.2)
< *I* / σ(*I*) >*	7.7 (0.8)
Completeness (%)*	94.9 (87.9)
Redundancy*	7.5 (7.2)
CC(1/2)	99.5 (42.7)
No. reflections	305313 (43622)
No. unique reflections	40753 (6083)
**Refinement**	
R_{work}/R_{free}	24.05/28.31
No. atoms	
Protein	8238
Ligand/ion	39
Water	119
Mean B factor (Å^2)	55.47
R.m.s deviations	
Bond lengths (Å)	0.013
Bond angles (º)	1.65
Ramachandran outlier (%)	0.4
Mol/AU	2

*Data in highest resolution shell are indicated in parenthesis. hydrol.: hydrolysis product.

Data availability

The atomic coordinates of SHP2(2-525) with the bound hydrolysis product of compound **SF12a** have been deposited in the Protein Data Bank under accession code 9TKT. All other data relevant to this work are available from authors on request.

Binding poses of SF10-12 and comparison to drug-like tunnel-targeting allosteric SHP2 inhibitor

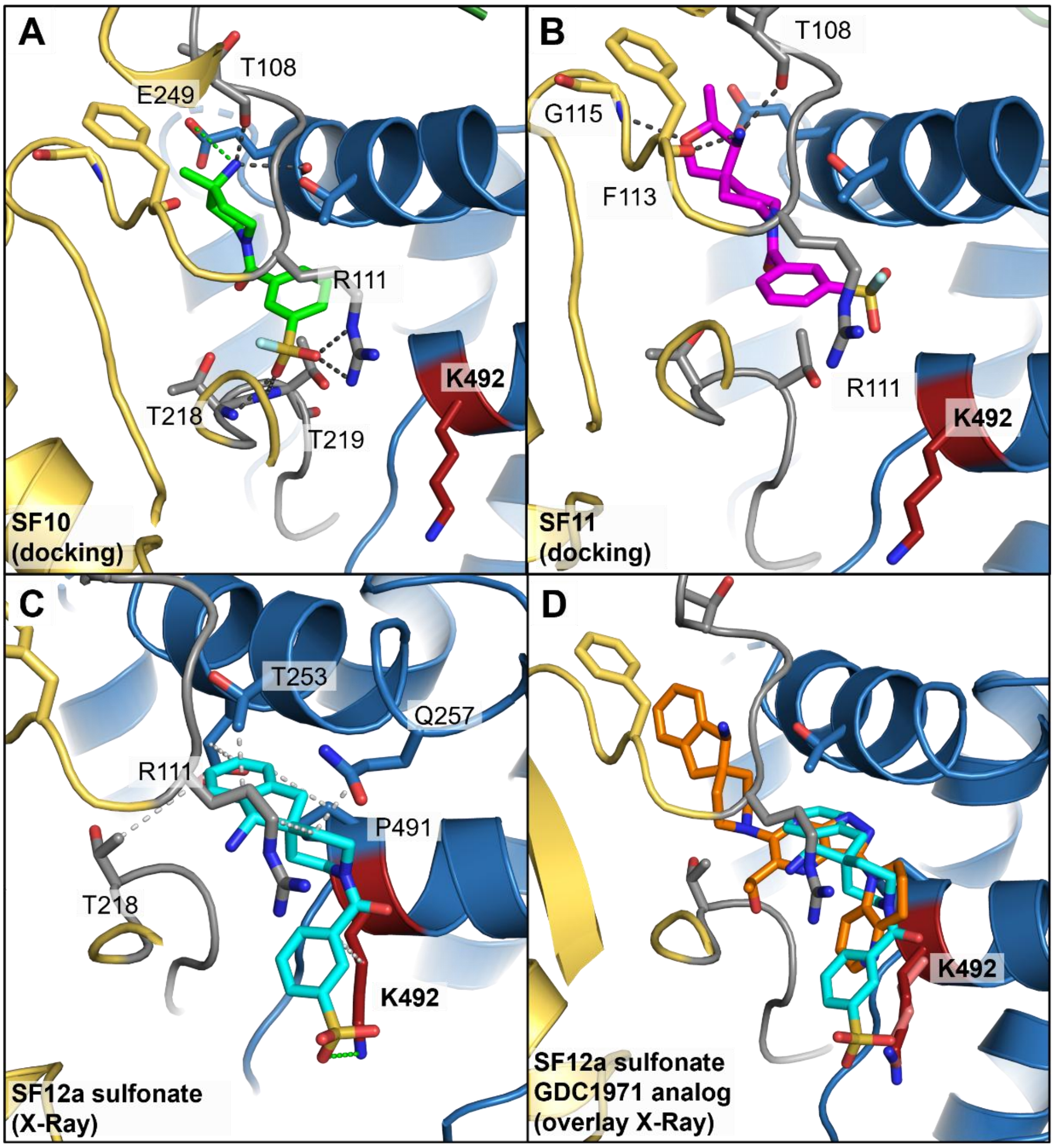

Figure S14: **A:** Non-covalent docking pose of **SF10** (green sticks) in PDB ID 6MDC. The distance between the S(VI) center and the ε-NH2 of K492 is 11.7 Å. Salt bridge is shows as green dashed line, H-bonds are shown as grey dashed lines. **B:** Non-covalent docking pose of **SF11** (pink sticks) in PDB ID 6MDC. The distance between the S(VI) center and the ε-NH2 of K492 is 10.5 Å. H-bonds are shown as grey dashed lines. **C:** X-Ray co-crystal structure of the aryl sulfonate hydrolysis product of **SF12a** (cyan sticks) formed during incubation in the co-crystallization buffer (PDB ID 9TKT). Hydrophobic interactions (shown as white dashed lines) between the aryl ring and the K492 side chain are observed as well as a salt bridge (shows as green dashed line) between the sulfonate and the side chain amine of K492. Additional hydrophobic contacts are observed between the spiro amine moiety and R111, T218, T253, Q257, and P491. **D**: Overlay of the obtained co-crystal structure of the sulfonate hydrolysis product of **SF12a** (cyan sticks), and of a structural analog of **GDC-1971** (orange sticks, PDB ID 8T6G). Both compounds feature the same (1*S*)-1-amino-1,3-dihydrospiro[indene-2,4'-piperidin]-1'-yl] amine substituent accommodated in different regions of the binding pocket. This shift facilitates ionic interactions between the aryl sulfonate of the **SF12a** hydrolysis product and K492 but positions the spiro amine in a non-optimal position for H-bond interactions seen for the **GDC1971 analog**.

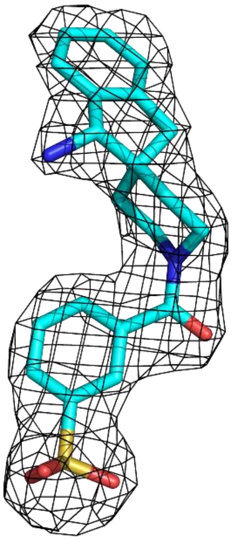

Figure S15: The aryl sulfonate hydrolysis product of compound **SF12a** is depicted with the surrounding electron density as a grey mesh to verify the compound position within the SHP2-**SF12a** hydrolysis product complex in the crystal structure.

Analysis of SF-reactive amino acids in co-crystal structures of allosteric tunnel site-targeting SHP2 inhibitors

Table S5: List of potentially nucleophilic amino acids within specified radius around the respective ligand in co-crystal X-ray structures of tunnel allosteric site targeting SHP2 inhibitors that feature the substructures that were included in the design of the biased fragment set. Residues were included if at least one side-chain heavy atom is located within the distance cutoff. Distances were measured in the respective co-crystal X-ray structures (prepared using Protein Preparation Wizard) in Maestro version 14.2.118, Release 2024-4). Residue pKa values were calculated using PROPKA[16]. n.a.: not applicable.

5 Å	8 Å	10 Å	pKa
**PDB ID 8T6G (RLY 1971 analog)[6]**			
H114	H114	H114	4.73
	H116	H116	6.65
	K129	K129	8.75
		K131	10.0
		K242	10.7
K244	K244	K244	10.9
K260	K260	K260	9.29
		Y263	10.6
K492	K492	K492	10.3
Y511	Y511	Y511	9.84
	Y515	Y515	9.84
**PDB ID 5EHR (SHP099)[3]**			
H114	H114	H114	4.99
		H116	6.82
		K129	9.26
		K244	8.66
	K260	K260	10.0
K492	K492	K492	10.3
Y511	Y511	Y511	9.84
	Y515	Y515	9.84
**PDB ID 7JVM (TNO155)[15]**			
H114	H114	H114	4.87
	H116	H116	6.59
		K129	9.10
		K244 is missing in this structure	n.a.
	K260	K260	10.1
		Y263	10.5
K492	K492	K492	10.2
Y511	Y511	Y511	9.84
	Y515	Y515	9.84

Table S6 List of labeled amino acids identified by tryptic digestion, along with the calculated pKa values for each labeled residue. The pKa values were estimated based on the co-crystal X-ray structures of tunnel-site targeting allosteric SHP2 inhibitors that feature the substructures that were included in the design of the biased fragment set. Residue pKa values were calculated using PROPKA[16].

Residue	pKa (PDB ID 8T6G, RLY 1971 analog)[6]	pKa (PDB ID 5EHR, SHP099)[3]	pKa (PDB ID 7JVM, TNO155)[15]
Y66	10.1	10.1	10.1
Y81	12.6	11.4	12.3
Y100	10.0	9.9	10.0
Y179	11.6	11.2	11.2
K198	10.0	10.9	11.3
K199	10.6	11.3	10.4
K235	11.2	11.2	11.2
K358	10.5	10.3	10.2
H426	5.7	5.9	5.9
K492	10.3	10.3	10.2
Y511	9.8	9.8	9.8

References

[1] S. Welte, K. H. Baringhaus, W. Schmider, G. Muller, S. Petry, N. Tennagels, "6,8-Difluoro-4-methylumbiliferyl phosphate: a fluorogenic substrate for protein tyrosine phosphatases", *Anal Biochem* **2005**, *338*, 32-38.

[2] L. Petri, P. Abranyi-Balogh, N. Csorba, A. Keeley, J. Simon, I. Randelovic, J. Tovari, G. Schlosser, D. Szabo, L. Drahos, G. M. Keseru, "Activation-Free Sulfonyl Fluoride Probes for Fragment Screening", *Molecules* **2023**, *28*.

[3] Y. N. Chen, M. J. LaMarche, H. M. Chan, P. Fekkes, J. Garcia-Fortanet, M. G. Acker, B. Antonakos, C. H. Chen, Z. Chen, V. G. Cooke, J. R. Dobson, Z. Deng, F. Fei, B. Firestone, M. Fodor, C. Fridrich, H. Gao, D. Grunenfelder, H. X. Hao, J. Jacob, S. Ho, K. Hsiao, Z. B. Kang, R. Karki, M. Kato, J. Larrow, L. R. La Bonte, F. Lenoir, G. Liu, S. Liu, D. Majumdar, M. J. Meyer, M. Palermo, L. Perez, M. Pu, E. Price, C. Quinn, S. Shakya, M. D. Shultz, J. Slisz, K. Venkatesan, P. Wang, M. Warmuth, S. Williams, G. Yang, J. Yuan, J. H. Zhang, P. Zhu, T. Ramsey, N. J. Keen, W. R. Sellers, T. Stams, P. D. Fortin, "Allosteric inhibition of SHP2 phosphatase inhibits cancers driven by receptor tyrosine kinases", *Nature* **2016**, *535*, 148-152.

[4] J. Garcia Fortanet, C. H. Chen, Y. N. Chen, Z. Chen, Z. Deng, B. Firestone, P. Fekkes, M. Fodor, P. D. Fortin, C. Fridrich, D. Grunenfelder, S. Ho, Z. B. Kang, R. Karki, M. Kato, N. Keen, L. R. LaBonte, J. Larrow, F. Lenoir, G. Liu, S. Liu, F. Lombardo, D. Majumdar, M. J. Meyer, M. Palermo, L. Perez, M. Pu, T. Ramsey, W. R. Sellers, M. D. Shultz, T. Stams, C. Towler, P. Wang, S. L. Williams, J. H. Zhang, M. J. LaMarche, "Allosteric Inhibition of SHP2: Identification of a Potent, Selective, and Orally Efficacious Phosphatase Inhibitor", *J Med Chem* **2016**, *59*, 7773-7782.

[5] J. T. Bagdanoff, Z. Chen, M. Acker, Y. N. Chen, H. Chan, M. Dore, B. Firestone, M. Fodor, J. Fortanet, M. Hentemann, M. Kato, R. Koenig, L. R. LaBonte, S. Liu, M. Mohseni, R. Ntaganda, P. Sarver, T. Smith, M. Sendzik, T. Stams, S. Spence, C. Towler, H. Wang, P. Wang, S. L. Williams, M. J. LaMarche, "Optimization of Fused Bicyclic Allosteric SHP2 Inhibitors", *J Med Chem* **2019**, *62*, 1781-1792.

[6] A. M. Taylor, B. R. Williams, F. Giordanetto, E. H. Kelley, A. Lescarbeau, K. Shortsleeves, Y. Tang, W. P. Walters, A. Arrazate, C. Bowman, E. Brophy, E. W. Chan, G. Deshmukh, J. B. Greisman, T. L. Hunsaker, D. R. Kipp, P. Saenz Lopez-Larrocha, D. Maddalo, I. J. Martin, P. Maragakis, M. Merchant, M. Murcko, H. Nisonoff, V. Nguyen, V. Nguyen, O. Orozco, C. Owen, L. Pierce, M. Schmidt, D. E. Shaw, S. Smith, E. Therrien, J. C. Tran, J. Watters, N. J. Waters, J. Wilbur, L. Willmore, "Identification of GDC-1971 (RLY-1971), a SHP2 Inhibitor Designed for the Treatment of Solid Tumors", *J Med Chem* **2023**, *66*, 13384-13399.

[7] G. M. Sastry, M. Adzhigirey, T. Day, R. Annabhimoju, W. Sherman, "Protein and ligand preparation: parameters, protocols, and influence on virtual screening enrichments", *J Comput Aided Mol Des* **2013**, *27*, 221-234.

[8] R. A. Friesner, J. L. Banks, R. B. Murphy, T. A. Halgren, J. J. Klicic, D. T. Mainz, M. P. Repasky, E. H. Knoll, M. Shelley, J. K. Perry, D. E. Shaw, P. Francis, P. S. Shenkin, "Glide: a new approach for rapid, accurate docking and scoring. 1. Method and assessment of docking accuracy", *J Med Chem* **2004**, *47*, 1739-1749.

[9] T. A. Halgren, R. B. Murphy, R. A. Friesner, H. S. Beard, L. L. Frye, W. T. Pollard, J. L. Banks, "Glide: a new approach for rapid, accurate docking and scoring. 2. Enrichment factors in database screening", *J Med Chem* **2004**, *47*, 1750-1759.

[10] K. Zhu, K. W. Borrelli, J. R. Greenwood, T. Day, R. Abel, R. S. Farid, E. Harder, "Docking covalent inhibitors: a parameter free approach to pose prediction and scoring", *J Chem Inf Model* **2014**, *54*, 1932-1940.

[11] M. Krug, M. S. Weiss, U. Heinemann, U. Mueller, "XDSAPP: a graphical user interface for the convenient processing of diffraction data usingXDS", *J Appl Crystallogr* **2012**, *45*, 568-572.

[12] A. J. McCoy, R. W. Grosse-Kunstleve, P. D. Adams, M. D. Winn, L. C. Storoni, R. J. Read, "Phaser crystallographic software", *J Appl Crystallogr* **2007**, *40*, 658-674.

[13] P. Emsley, B. Lohkamp, W. G. Scott, K. Cowtan, "Features and development of Coot", *Acta Crystallogr D Biol Crystallogr* **2010**, *66*, 486-501.

[14] G. N. Murshudov, A. A. Vagin, E. J. Dodson, "Refinement of macromolecular structures by the maximum-likelihood method", *Acta Crystallogr D Biol Crystallogr* **1997**, *53*, 240-255.

[15] M. J. LaMarche, M. Acker, A. Argintaru, D. Bauer, J. Boisclair, H. Chan, C. H. Chen, Y. N. Chen, Z. Chen, Z. Deng, M. Dore, D. Dunstan, J. Fan, P. Fekkes, B. Firestone, M. Fodor, J. Garcia-Fortanet, P. D. Fortin, C. Fridrich, J. Giraldes, M. Glick, D. Grunenfelder, H. X. Hao, M. Hentemann, S. Ho, A. Jouk, Z. B. Kang, R. Karki, M. Kato, N. Keen, R. Koenig, L. R. LaBonte, J. Larrow, G. Liu, S. Liu, D. Majumdar, S. Mathieu, M. J. Meyer, M. Mohseni, R. Ntaganda, M. Palermo, L. Perez, M. Pu, T. Ramsey, J. Reilly, P. Sarver, W. R. Sellers, M. Sendzik, M. D. Shultz, J. Slisz, K. Slocum, T. Smith, S. Spence, T. Stams, C. Straub, V. Tamez, Jr., B. B. Toure, C. Towler, P. Wang, H. Wang, S. L. Williams, F. Yang, B. Yu, J. H. Zhang, S. Zhu, "Identification of TNO155, an Allosteric SHP2 Inhibitor for the Treatment of Cancer", *J Med Chem* **2020**, *63*, 13578-13594.

[16] M. H. Olsson, C. R. Sondergaard, M. Rostkowski, J. H. Jensen, "PROPKA3: Consistent Treatment of Internal and Surface Residues in Empirical pKa Predictions", *J Chem Theory Comput* **2011**, *7*, 525-537.
